# Aberrant phase separation of two PKA RIβ neurological disorder mutants leads to mechanistically distinct signaling deficits

**DOI:** 10.1016/j.celrep.2025.115797

**Published:** 2025-06-11

**Authors:** Emily H. Pool, Alexander Glebov-McCloud, Ha Neul Lee, Julia C. Hardy, Valeria Pane, Friedrich W. Herberg, Susan S. Taylor, Sohum Mehta, Stefan Strack, Jin Zhang

**Affiliations:** 1 Department of Pharmacology, University of California, San Diego, La Jolla, CA 92093, USA; 2 Department of Chemistry and Biochemistry, University of California, San Diego, La Jolla, CA 92093, USA; 3 Department of Neuroscience and Pharmacology, Iowa Neuroscience Institute, University of Iowa, Iowa City, IA 52242, USA; 4 Shu Chien-Gene Lay Department of Bioengineering, University of California, San Diego, La Jolla, CA 92093, USA; 5 Department of Biochemistry, University of Kassel, 34132 Kassel, Germany; 6 Moores Cancer Center, University of California, San Diego, La Jolla, CA 92093, USA; 7 These authors contributed equally; 8 Lead contact

## Abstract

Spatiotemporal regulation of key-node signaling molecules, such as 3′,5′-cyclic adenosine monophosphate (cAMP)-dependent protein kinase (PKA), is critical for normal cell physiology and susceptible to dysregulation in disease. Liquid-liquid phase separation (LLPS) is broadly recognized as a fundamental component of signal regulation, yet the connections between physiological and disease-linked biomolecular condensates are not well understood. Here, we show that an understudied, brain-specific PKA regulatory subunit, RI**β**, forms biomolecular condensates with distinct features from the ubiquitous isoform, RI**α**. We demonstrate that two RI**β** mutants linked to neurodegenerative (L50R) or neurodevelopmental (R335W) pathologies produce aberrant condensates that trap the PKA catalytic subunit within a gel-like matrix or cAMP-insensitive holoenzyme complex, respectively. RI**β**^L50R^ condensates, in particular, lead to disrupted spatiotemporal control of PKA signaling and diminished PKA activity, resulting in phenotypic hallmarks of neurodegeneration. Our work highlights the functional importance of biomolecular condensates and the critical link between dysregulated LLPS and neurological disorders.

## In brief

Pool et al. demonstrate PKA RI**β** phase separation and evaluate how its condensates are dysregulated by two neurological disease mutations. Both Marbach-Schaaf neurodevelopmental syndrome variant R335W and NLPD-PKA neurodegenerative variant L50R sequester PKA-C**α** and pathologically impair downstream signaling, albeit via different mechanisms consistent with their distinct phenotypes.

## Graphical abstract

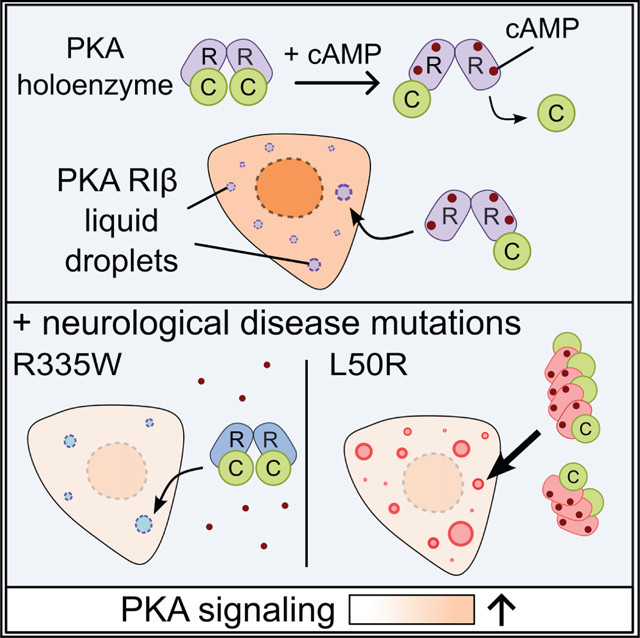

## INTRODUCTION

A fundamental role of biochemical activity architectures is the faithful transduction of extracellular cues via intracellular signals. cAMP-dependent protein kinase (PKA) is a master regulator of signaling, coordinating myriad functional outcomes including synaptic plasticity ^1,2^ and learning and memory. ^3,4^ Aberrant PKA signaling is associated with Alzheimer’s (AD), Parkinson’s (PD), and Huntington’s diseases, ^5–14^ but the underlying molecular mechanisms are poorly understood. The PKA holoenzyme consists of a heterotetramer of two catalytic (C) subunits and a regulatory (R) subunit dimer. cAMP activates the holoenzyme by binding tandem cyclic nucleotide binding (CNB) domains on each R subunit, triggering intricate conformational changes that unleash the C subunit. ^15–17^ Each of the four R subunit isoforms (RIα, RIβ, RIIα, and RIIβ) comprises a distinct molecular machine with different expression patterns, interaction partners, and functions. ^18,19^ Recent work has increasingly focused on the less-studied RI isoform, RIβ. ^20–22^ Deleting RIβ causes severe learning and memory deficits in mice, ^23,24^ and RIβ knockdown in cultured primary neurons results in decreased phosphorylation of cAMP response element binding protein (CREB), a transcription factor known to be phosphorylated in a PKA-dependent manner to regulate learning and memory. ^22,25^ Despite these studies, it is not clear how the unique biophysical properties of RIβ tune this molecular machine to specifically contribute to the PKA signaling landscape.

Recently, three heterozygous mutations in *PRKAR1B* were identified as causative for symptoms associated with a neurodevelopmental disorder including insensitivity to pain and global developmental delay. ^26^ The most prevalent mutation, RIβ^R335W^, has since seen doubling in the number of unrelated cases for the so-dubbed Marbach-Schaaf neurodevelopmental syndrome (MASNS, OMIM #619680). ^27^ R335 is critically involved in stabilizing cAMP binding to the C-terminal CNB domain, CNB-B, ^16,28–30^ but how this mutation affects RIβ biophysical properties and signaling is unknown. Meanwhile, individuals carrying an RIβ L50R mutation exhibit dementia with parkinsonism and neuronal loss, with immunohistology of hippocampal slices showing aggregate-like RIβ^L50R^ assemblies. ^31^ L50 is part of the N-terminal docking and dimerization domain (D/D), which also functions as the docking interface for A-kinase anchoring proteins (AKAPs) that subcellularly localize PKA and promote signal specificity. ^32–34^ Although the RIβ^L50R^ inclusions have been linked to this disease, known as neuronal loss and parkinsonism driven by a PKA mutation (NLPD-PKA), the mechanistic connections between RIβ^L50R^ “aggregates” and disease pathology remain unclear. ^35^

Liquid-liquid phase separation (LLPS) of macromolecules, a process by which molecular components de-mix into condensed and dilute states, has emerged as a principal organizer of cellular biochemical activity architectures,^36–40^ giving rise to biomolecular condensates involved in regulating a variety of processes, including signal transduction.^41–44^ Intriguingly, several proteins that are found in pathological aggregates or inclusions in neurodegenerative diseases, such as α-synuclein,^45,46^ FUS,^47,48^ and tau,^5,49,50^ can undergo LLPS, and dysregulation of these condensates is linked to their respective disease states. As biomolecular condensates exhibit a wide array of properties, a critical question is how pathological assemblies are linked to their physiological counterparts and how abnormal functions emerge from aberrant condensates.

Previously, we discovered that RIα can undergo LLPS to form biomolecular condensates that dynamically buffer cAMP and PKA signaling to maintain pathway specificity, and that disruption of RIα condensates can trigger pathological signaling. ^51,52^ Here we show that wild-type RIβ also undergoes cAMP-dependent LLPS, mediated by its dimerization interfaces and flexible disordered linker, to form biomolecular condensates that recruit the PKA C subunit but otherwise show distinct features from RIα condensates. Notably, the disease-linked R335W and L50R mutations induce aberrant RIβ assemblies, resulting in mechanistically distinct “trapping” of the C subunit that is critical to their detrimental effects. Our work highlights the general role of LLPS as a critical organizer of PKA signaling and the exquisite specificity of each isoform as distinct molecular machines. Furthermore, this work provides invaluable mechanistic insight into the etiologies of NLPD-PKA and MASNS.

## RESULTS

### PKA RIβ forms biomolecular condensates in cells

**Figure 1.**
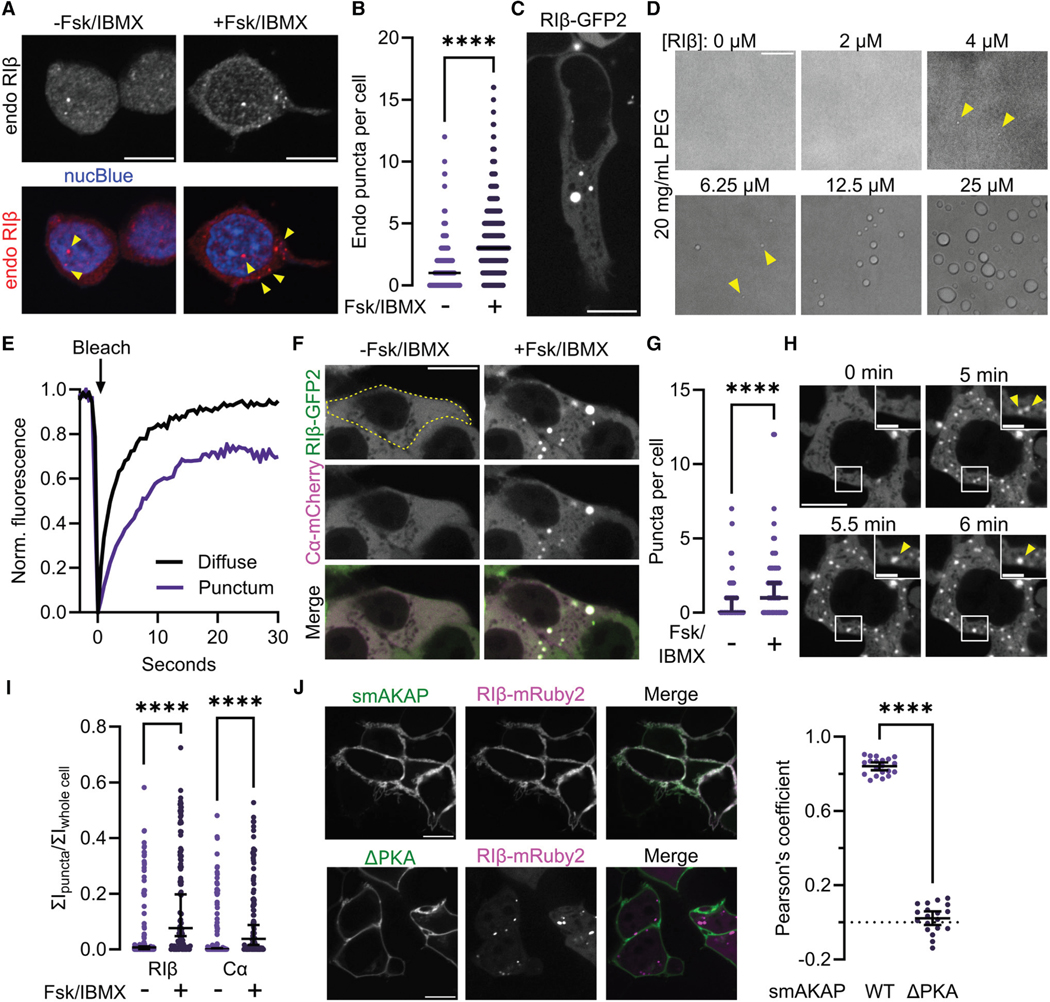
RIβ undergoes liquid-liquid phase separation (A) Representative immunofluorescence images of endogenous RIβ puncta in PC12 cells pre(−) and post(+) 30-min Fsk/IBMX treatment. (B) Quantification of endogenous puncta per cell in immunofluorescence experiments. *n*_− Fsk/IBMX_ = 622, *n*_+Fsk/IBMX_ = 778 cells; ****p < 1 × 10^−15^, Welch’s unpaired t test. (C) Representative image of a HEK293T cell overexpressing RIβ-GFP2 showing punctate assemblies. (D) *In vitro* phase separation assays of purified RIβ suggest a 4 μM threshold concentration for assembly formation. Contrast in 4 μM image enhanced for clarity. (E) Representative recovery of photobleached RIβ-GFP2. (F) Representative images of HEK293T cells co-expressing RIβ-GFP2 and Cα-mCherry pre(−) and post(+) 20-min cAMP stimulation, showing formation of co-localized puncta. (G) Quantification of RIβ-GFP2 puncta from (F). *n* = 70 cells; ****p = 2.07 × 10^−6^. (H) Representative time-lapse showing fusion of newly formed RIβ puncta (experiment as in F). (I) Partition coefficients of RIβ-GFP2 and Cα-mCherry in puncta. (J) smAKAP recruitment inhibits RIβ condensates. ****p < 1 × 10^−15^; *n*_smAKAP_ = 20, *n*_L2P2_ = 19 whole images from three independent experiments. Scale bars, 10 μm; insets in (H), 3 μm. In (A), (D), and (H), puncta are indicated with yellow arrowheads; dashed lines in (F) mark cell borders. Dot plots in (B), (G), and (I) show median ± 95% CI and significance determined by Wilcoxon test, and in (J), mean ± 95% CI, Welch’s unpaired t test. See also Figure S1.

We first investigated whether endogenously expressed RIβ exhibits puncta. RIβ immunofluorescence staining revealed discrete puncta in PC12 cells (95% confidence interval [CI]: 1− 1; *n* = 622 cells) (Figures 1A and 1B, S1A). Similar puncta were observed in HEK293T cells overexpressing V5-tagged RIβ (Figure S1B). We then examined the dynamics of RIβ in live cells. GFP2-tagged RIβ formed highly dynamic, spherical assemblies in HEK293T cells (95% CI: 2− 3; *n* = 87 cells) (Figure 1C), which visibly sampled the surrounding cellular milieu on the seconds timescale (Video S1). Similar behaviors were observed using RIβ fused to mRuby2, and in PC12 and HeLa cells expressing RIβ-GFP2, whereas control cells expressing EGFP alone showed only diffuse fluorescence (Figures S1C–S1F). Purified RIβ protein also formed assemblies *in vitro* at concentrations as low as 4 μM in a minimal reconstitution system, which increased in size and were observed to fuse and wet the glass surface at higher concentrations (Figures 1D and S1G), suggesting that the phenomenon observed in cells was driven by RIβ. Finally, to probe the biophysical properties of cellular RIβ puncta, we performed fluorescence recovery after photo bleaching (FRAP) analyses in HEK293T cells expressing RIβ-GFP2, which revealed that tagged RIβ can dynamically exchange between the puncta and diffuse pools (Figures 1E, S1H–S1J). These data indicate that RIβ forms membrane-less biomolecular condensates with liquid-like behaviors, reminiscent of RIα condensates formed via LLPS. ^51^

In the classical model, the PKA holoenzyme exists in dynamic equilibrium between R:C bound and unbound states, mediated by cAMP binding to the R-subunits, which diminishes R:C interactions.^15–17^ We previously showed that PKA Cα inhibits RIα condensate formation, and that cAMP relieves this inhibition.^51^ To test whether RIβ condensates are similarly influenced by Cα and cAMP, we co-overexpressed RIβ-GFP2 and Cα-mCherry in HEK293T cells. Indeed, Cα co-expression decreased the number of RIβ puncta (95% CI_median_ : 0–1, *n* = 70 cells, *p* = 5.83 × 10^−11^), whereas stimulation with the adenylyl cyclase (AC) activator forskolin (Fsk) and phosphodiesterase (PDE) inhibitor 3-isobutyl-1-methylxanthine (IBMX) partially relieved this inhibition (95% CI_basal_ : 0−1, 95% CI_cAMP-stimulated_ : 1−2, *n* = 70 cells, *p* = 2.07 × 10^−6^ ) (Figures 1F and 1G). Cα-mCherry expressed alone did not form puncta, confirming that Cα does not drive RIβ condensate formation (Figure S1K). Newly formed puncta coalesced on the minute timescale following cAMP stimulation (Figure 1H), highlighting the liquid-like properties of these assemblies. To verify the cAMP effect at endogenous RIβ concentrations, we compared the distribution of RIβ immunofluorescence in PC12 cells under basal and Fsk/IBMX-stimulated conditions and observed a nearly 3-fold increase in endogenous puncta in Fsk/IBMX-stimulated cells (95% CI: 3−3, *n* = 778 cells, *p* < 1 × 10^−15^ ) (Figures 1A and 1B). Additionally, an RIβ-tethered cAMP sensor, G-Flamp1,^53^ displayed elevated fluorescence and reduced response to cAMP stimulation within puncta compared with the diffuse pool in RIα knockout (KO) HEK293T cells (Figures S1L and S1M), consistent with cAMP accumulation by RIβ condensates. Yet despite the high cAMP levels induced by AC activation and PDE inhibition (Figure S1N), we found that Cα was retained by RIβ puncta in HEK293T cells co-overexpressing RIβ-GFP2 and Cα-mCherry, and that the partitioning of both species in the puncta increased in response to cAMP stimulation (RIβ: 95% CI_basal_ : 0.00134–0.0107, 95% CI_+cAMP_ : 0.0478–0.198, Cα: 95% CI_basal_ : 0.000–0.00417, 95% CI_+cAMP_ : 0.0163–0.0876, *n* = 101 cells, *p*_RIβ_ < 1 × 10^−15^, *p*_Cα_ = 3.7 × 10^−14^ ) (Figures 1F and 1I, S1O). Our findings contradict the canonical model of full R:C dissociation at high [cAMP], similar to recent work showing that RIα condensates contain a non-canonical PKA holoenzyme that both retains Cα and is catalytically active.^15,54,55^

AKAPs shape PKA signaling specificity by subcellularly localizing the holoenzyme through interactions with the R subunit D/D domain interface. We wondered if this binding interaction and RI localization would affect RIβ LLPS, as AKAP recruitment inhibits RIα condensate formation. ^55^ To answer this question, we selected a representative AKAP, small membrane (sm)AKAP, which anchors RI holoenzymes to the plasma membrane, ^56^ and co-expressed GFP-tagged smAKAP with RIβ-mRuby2. Indeed, RIβ^WT^ was recruited by smAKAP to the plasma membrane, abolishing cytosolic condensates, whereas mutations in the smAKAP PKA binding motif (ΔPKA) ^34^ prevented recruitment and restored condensate formation (Figure 1J). In sum, our studies suggest that RIβ forms biomolecular condensates via LLPS, dictated by C subunit interactions, AKAP binding, and cAMP dynamics.

### Diverse condensate behaviors of RIβ deletion mutants

**Figure 2.**
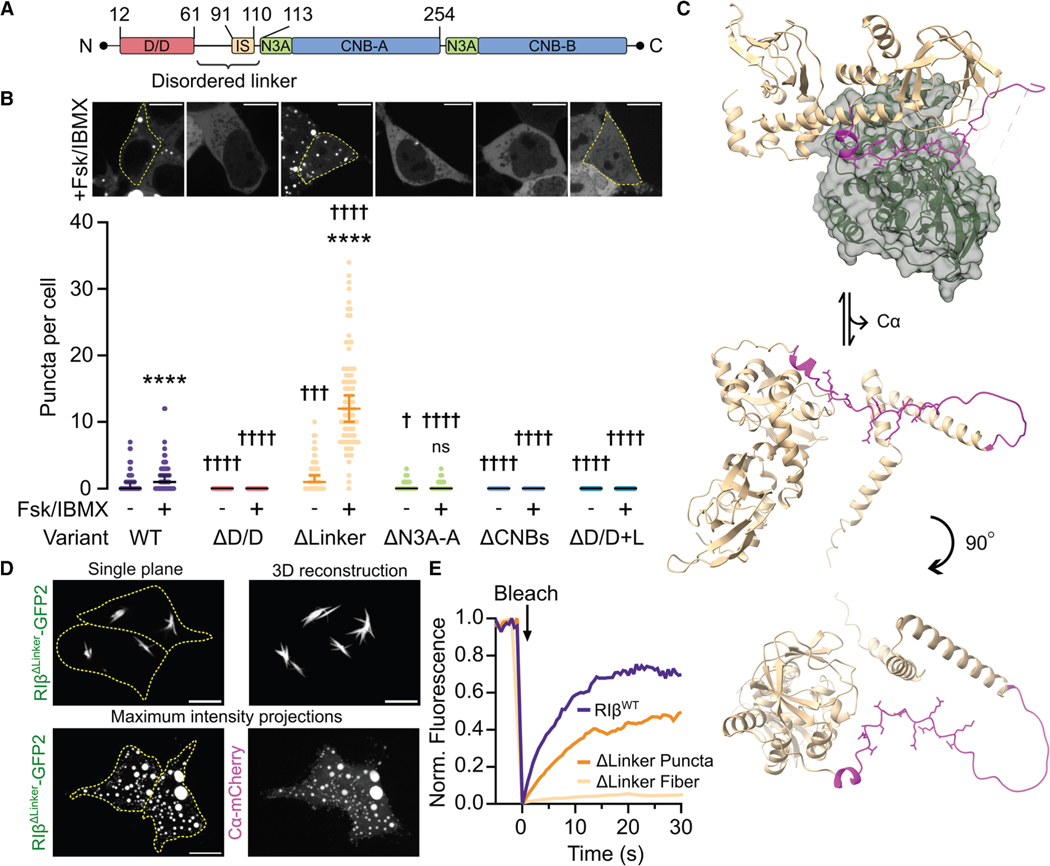
Domain deletions reveal RIβ-specific characteristics for phase separation (A) Domain architecture of RIβ. (B) Puncta quantification of GFP2-tagged RIβ domain deletion mutants co-expressed in HEK293T with Cα-mCherry ± cAMP stimulation. Representative post-stimulation images shown. Statistical significance is ±Fsk/IBMX (asterisks, paired Student’s t tests) or vs. wild-type of the same condition (daggers, Mann-Whitney test). ††††*p*_ΔD/D−cAMP_ = 2.04 × 10^−12^, ††††*p*_ΔD/D+cAMP_ < 1 × 10^−15^, †††*p*_ΔLinker−cAMP_ = 9.12 × 10^−4^, ††††*p*_ΔLinker+cAMP_ < 1 × 10^−15^, ****p_ΔLinker_ < 1 × 10^−15^, †*p*_ΔN3A-A− cAMP_ = 0.0331, ††††*p*_ΔN3A-A+cAMP_ = 4.44 × 10^−7^, *p*_ΔN3A-A±cAMP_ = 0.531, ††††*p*_ΔCNBs−cAMP_ = 4.66 × 10^−13^, ††††*p*_ΔCNBs +cAMP_ < 1 × 10^−15^, ††††*p*_ΔD/D+L−cAMP_ = 1.25 × 10^−10^, ††††*p*_ΔD/D+L+cAMP_ = 2 × 10^−15^. Dot plots show median ± 95% CI. (C) Structural model showing increased disorder of the linker region when not bound to Cα. RIβ ribbon shown in tan with the most disordered residues in magenta and IS showing side chains, Cα shown in green ribbon with space-fill. Top: 4DIN, bottom: AlphaFold2 structural prediction of full-length human RIβ. (D) Representative slice and 3D reconstruction of singly expressed RIβ^ΔLinker^-GFP2 (top) and representative maximum intensity projections of co-expressed RIβ^ΔLinker^-GFP2 and Cα-mCherry (bottom) in RIα KO HEK293T cells. Brightness automatically adjusted for 3D reconstruction and Cα-mCherry final projection for visual clarity. (E) Recovery curves of photobleached RIβ^ΔLinker^ condensates. For (B) and (E), WT data reproduced from Figure 1. Scale bars in (B) and (D), 10 μm. Dashed lines indicate cell borders. See also Figure S2.

The formation of RI dimers is mediated by two primary interaction sites: the D/D domain and a lower affinity N3A motif at the N terminus of CNB-A (N3A-A) (Figure 2A).^32,57–59^ We hypothesized that these interfaces contribute to RIβ LLPS because multivalency contributes to LLPS in many systems.^38,60–62^ To probe the roles of these dimerization sites in RIβ LLPS, we generated RIβ truncation mutants and monitored their ability to form puncta when overexpressed (Figure S2A). Consistent with its requirement for RIα LLPS,^51^ deleting the D/D domain (RIβ^ΔD/D^ ) abolished RIβ puncta formation, even upon cAMP stimulation (*n* = 67 cells) (Figure 2B). Overexpressing a construct encoding only the D/D domain (RIβ^D/D^ ) also did not produce puncta, indicating the D/D domain is necessary but insufficient for RIβ LLPS (Figures S2B and S2C). Similarly, deleting the N3A-A motif (RIβ^ΔN3A-A^ ) almost completely abrogated phase separation (95% CI_±Fsk/IBMX_ : 0–0, *n* = 68 cells) (Figure 2B). These data indicate that RIβ LLPS requires both dimerization sites.

The CNB domains are critical to PKA activation, as cAMP binding to these regions triggers the conformational changes that unleash the C subunit. ^15–17^ The CNB domains also make physical contact with the C subunit, with distinct quaternary structures for each RI. ^21^ We therefore asked if the CNB domains are required for RIβ LLPS. No puncta were observed in basal or cAMP-stimulated HEK293T cells co-expressing Cα-mCherry and a GFP2-tagged RIβ mutant lacking both CNB domains (RIβ^ΔCNB^, *n* = 72 cells) (Figure 2B), in contrast to RIα, where a similar truncation mutant partially retained LLPS capability. ^51^ Meanwhile, a construct expressing only the RIβ CNB domains (RIβ^ΔD/D+L^ ) also showed no puncta formation (*n* = 59 cells) (Figure 2B). Together, these data indicate that the CNB domains are also required for RIβ LLPS.

In RI subunits, the D/D and the CNB domains are connected by a disordered linker region that contains a pseudosubstrate for the C subunit, which is buried within the C subunit active site cleft in the holoenzyme^17,63^ (Figures 2C and S2D). Upon cAMP binding, the linker is thought to become more exposed and disordered, accounting for the lack of N-terminal electron density in published RI subunit crystal structures^15,21^ (Figure 2C). We previously showed that deleting the RIα disordered linker region abolishes LLPS.^51^ Because the RIβ linker also contains a high proportion of disorder-promoting residues (73.3% in the linker vs. 63.0% across full-length RIβ) (Figures S2E and S2F), we anticipated that loss of the RIβ linker would lead to decreased LLPS, given that intrinsic disorder is a key driver of phase separation.^38,64–66^ However, deleting the RIβ linker region induced aberrant condensate behaviors. Unexpectedly, we observed fiber-like structures in HEK293T cells overexpressing the linker-deletion mutant, RIβ ^ΔLinker^-GFP2 (Figure 2D), whereas a construct encoding only the linker region showed diffuse fluorescence (Figures S2B and S2C). FRAP measurements of RIβ^ΔLinker^ fibrillar assemblies demonstrated slow and incomplete recovery, indicating that RIβ^ΔLinker^ cannot freely exchange with the diffuse pool (Figures 2E, S2G, and S2H). This suggests the linker region may provide conformational flexibility and promote liquid-like properties in RIβ^WT^ condensates, which were not observed to mature into fibers. Interestingly, Cα co-expression decreased fiber formation. Instead, cAMP-responsive spherical puncta were observed in cells co-expressing RIβ^ΔLinker^ - GFP2 and Cα-mCherry (95% CI_basal_ : 1−2, 95% CI_+cAMP_ : 10−14 puncta per cell, *n* = 81 cells), with greater puncta numbers per cell in both basal and cAMP-stimulated conditions compared with RIβ^WT^ (*p*_basal_ = 0.000912, *p*_cAMP_ < 1 × 10^−15^ ) (Figures 2B and 2D). These data indicate that the RIβ^ΔLinker^ mutant retains cAMP-dependence and C subunit binding despite loss of the inhibitory site (IS), presumably through interactions between Cα and the CNB domains.^21^ Additionally, FRAP experiments showed that RIβ^ΔLinker^ can dynamically exchange between puncta and diffusible pools in the presence of co-expressed Cα (Figures 2E and S2H). Thus, RIβ^ΔLinker^ appears capable of forming both liquid- and solid-like assemblies depending on the presence of Cα. Collectively, our results highlight the unique features of RIβ LLPS compared with its more ubiquitous counterpart, RIα, and reveal a complex role for the RIβ-disordered linker.

### A neurodevelopmental disorder mutation eliminates cAMP-dependent phase separation

**Figure 3.**
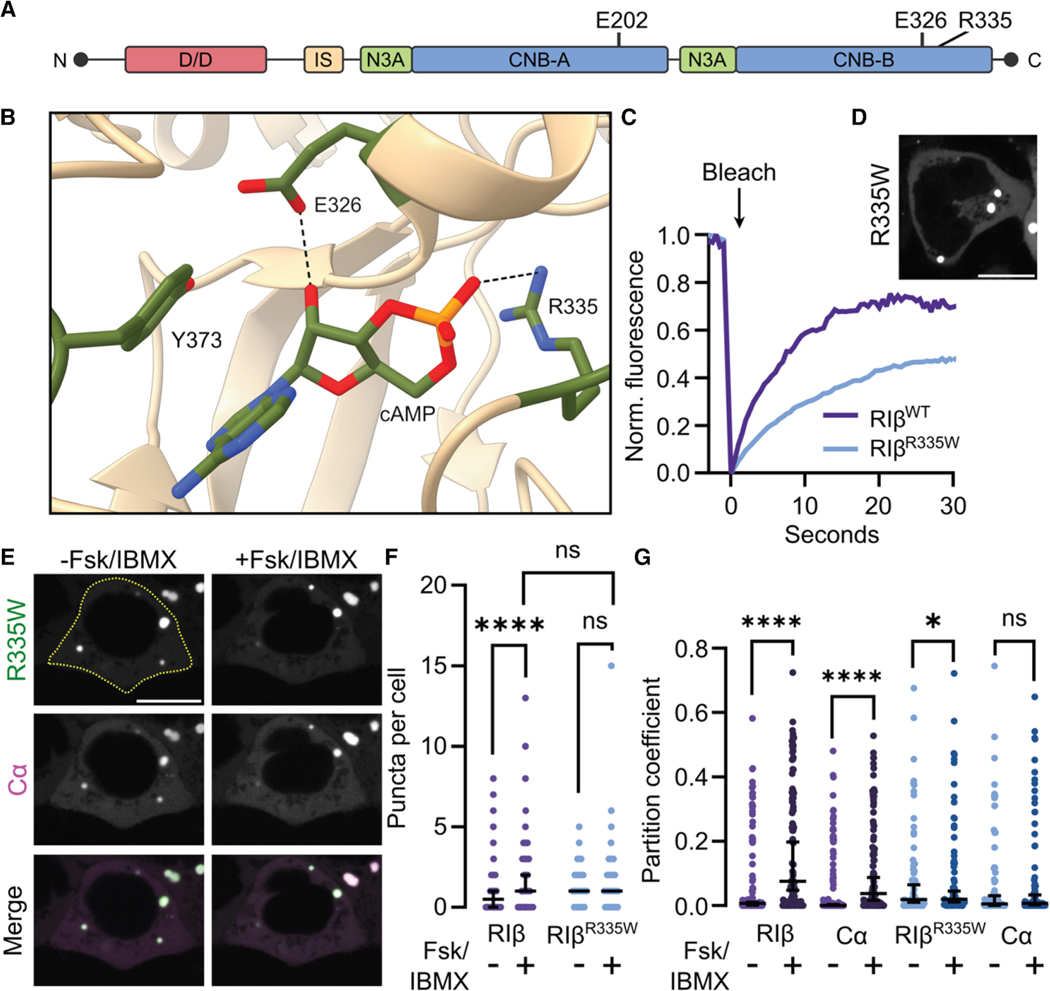
The RIβ^R335W^ neurodevelopmental mutation abolishes cAMP-stimulated LLPS (A) Critical residues in the CNB cassettes. (B) Structure of the CNB-B cAMP binding cassette using the cAMP-bound RIα structure PDB: 4MX3. (C) Representative recovery curve of photobleached RIβ^R335W^ condensates. (D) Representative image of HEK293T cells singly expressing RIβ ^R335W^-GFP2. (E) Representative images of cells co-expressing RIβ^R335W^-GFP2 with Cα-mCherry before (−) and 20 min after (+) cAMP stimulation. Dashed line indicates cell borders. (F and G) Puncta quantification (F) and partition coefficients (G) from cells as in (E), compared with wild-type. Puncta quantification: *n*_R335W_ = 150 cells, *p*_R335W±cAMP_ = 0.623; Wilcoxon test. *p*_WT/R335W+cAMP_ = 0.475; Mann-Whitney test. Partition coefficients: *n*_R335W_ = 72 cells, 95% CI_R335W−cAMP_ = 0.0107–0.0650, 95% CI_R335W+cAMP_ = 0.0115–0.0457, *p = 0.0300, 95% CI_Cα−cAMP_ = 0.000–0.00313, 95% CI_Cα+cAMP_ = 0.00235–0.0337, *p* = 0.148; paired Wilcoxon tests. WT data in (C), (F), and (G) reproduced from Figure 1. Scale bars in (D) and (E), 10 μm. Dot plots, median ± 95% CI. Overexpression conducted in HEK293T cells. See also Figure S3.

Among the first diseases causally associated with RIβ, MASNS is a neurodevelopmental disorder characterized by developmental delay, intellectual disability, and high pain tolerance.^26,27^ A single monoallelic and *de novo* point mutation in RIβ, encoding an R335W substitution in the C-terminal cAMP binding pocket, was found to be causative for 11 of 13 cases in unrelated children (Figures 3A and 3B).^26,27^ However, the impact of the R335W mutation on RIβ LLPS and its mechanistic link to MASNS are un- explored. When we expressed GFP2-tagged RIβ ^R335W^ alone in HEK293T cells, we observed similar puncta numbers, with largely liquid-like properties, but reduced apparent diffusion, compared with RIβ WT (*p*_mobile fraction_ = 0.0903, *p*_*Dapp*_ = 1.39 × 10^−10^, *n* = 43 puncta) (Figures 3C, 3D, and S3A–S3C). RIβ^R335W^ and RIβ^WT^ also formed mixed condensates (Figure S3D). Interestingly, Cα-GFP2, found in either RIβ^R335W^ condensates (Figure 3E) or mixed condensates (Figure S3D), showed limited exchange with the cytosol (Figure S3E).

RIβ^R335W^ is expected to exhibit impaired cAMP binding. Similar mutations incompletely prevent cAMP binding to the RIα CNB-B^30,67^ by eliminating the R335-cAMP hydrogen bond, decreasing cAMP stability within the pocket. To clarify if cAMP-induced RIβ phase separation is affected by this neurodevelopmental mutation, we quantified the number of RIβ^R335W^-containing condensates in cells co-overexpressing GFP2-labeled RIβ^R335W^ with Cα-mCherry. No change was observed upon stimulation (95% CI_basal_ : 1− 1; 95% CI_cAMP_ : 1−1 puncta per cell, *p =* 0.623, *n* = 150 cells) (Figures 3E and 3F). This trend was echoed in the partition coefficients of RIβ^R335W^ and Cα, where Fsk/IBMX stimulation did not increase RIβ^R335W^ or Cα partitioning (*p*_R335W_ = 0.0300 (basal partition > cAMP-stimulated), *p*_Cα_ = 0.148, *n* = 72 cells), unlike the dramatic cAMP-induced increase we observed with RIβ^WT^ plus Cα (Figure 3G). These data collectively suggest the R335W mutation disrupts cAMP-dependent RIβ LLPS but not intrinsic LLPS propensity.

The loss of cAMP-induced RIβ^R335W^ LLPS prompted us to probe the specific role of cAMP binding in RIβ LLPS. To do this, we introduced two mutations, E202A and E326A, that destabilize cAMP binding to CNB-A and CNB-B, respectively ^29,68^ (Figures 3A and 3B). A similar mutation, E326Q, reduced cAMP binding in RIα by 280-fold.^69^ RIβ^E202A^ and RIβ^E326A^ displayed weak cAMP-dependent LLPS despite largely unaltered LLPS capacity when expressed without Cα (Figures S3F–S3I). Other well-studied point mutations within the CNB pockets, R211K and R335K, also did not abolish cAMP-dependent phase separation (Figure S3J),^30,67^ suggesting residual cAMP binding occurs at high cAMP concentrations. Only when we mutated both sites (“ΔcAMP”) to block cAMP binding did we observe a loss of cAMP-dependent puncta formation similar to RIβ^R335W^-expressing cells (Figures S3G and S3K). Expressing RIβ^ΔcAMP^ alone produced a similar number of puncta to RIβ^WT^ (Figure S3L), indicating that the double mutation specifically blocks cAMP-dependent LLPS. Thus, cAMP-dependent RIβ LLPS depends on the functionality of both CNB domains. Moreover, the differences observed between these mutants highlight the particularly strong effect of the disease-relevant R335W mutant on disrupting cAMP-dependent phase separation.

### R335W impairs PKA activity

**Figure 4.**
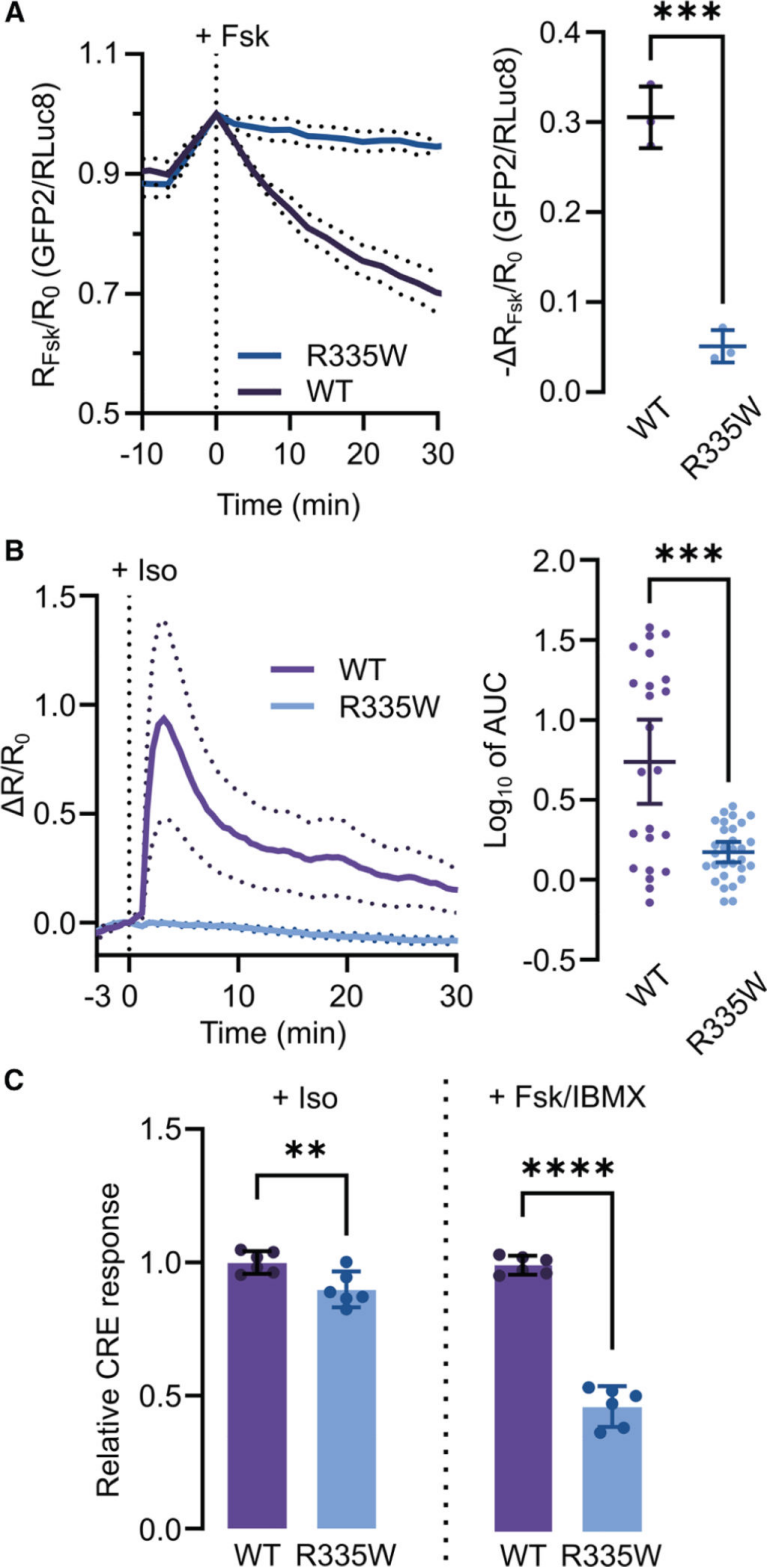
The Marbach-Schaaf R335W mutation is dominant negative and dysregulates PKA signaling by sequestering the catalytic subunit (A) Loss of R:C BRET emission ratios upon 50 μM forskolin stimulation in cells overexpressing RIβ^WT^ - or RIβ^R335W^ -RLuc8 and GFP2-Cα. ***p = 0.000340. (B) PKA activity response curves and area under the curve following stimulation with 5 μM Iso in cells overexpressing ExRai-AKAR2 and RIβ-mRuby2. *n*_WT_ = 23, *n*_R335W_ = 30 cells, ***p = 2.26 × 10^−4^. (C) Normalized CRE transcription in cAMP-stimulated cells expressing RIβ^WT^ - or RIβ ^R335W^ -mRuby2. **p_Iso_ = 0.00814 and ****p_Fsk/IBMX_ = 1.60 × 10^−8^. Experiments conducted in RIα KO HEK293T and compared by unpaired Student’s or Welch’s t tests. Data in (A), mean ± SD, others, mean ± 95% CI. See also Figure S4.

Although defective cAMP binding is expected to lead to deficits in PKA activity, RIβ ^R335W^ was reported not to significantly alter the total, or cAMP-stimulated PKA activity measured in lysates from HEK293 cells expressing RIβ^WT^ or RIβ^R335W^. ^26^ In addition, previous work indicated that type I PKA holoenzyme can be activated through a single intact CNB.^28,67,70,71^ To further characterize this mutant, we examined the R:C dissociation and signaling capability of RIβ^R335W^ with respect to other CNB mutations. We chose RIα KO HEK293T cells ^51^ for these experiments to avoid the influence of endogenous RIα, which is transcribed at copy numbers 3-fold higher than RIβ in HEK293, ^72^ allowing overexpressed tagged RIβ to mimic the 3.3:1 endogenous RI:C ratio. ^73^ First, we conducted bioluminescence resonance energy transfer (BRET) assays using RIβ-Rluc8 and GFP2-Cα. ^74–77^ This assay measures energy transfer between a luminescent donor (RLuc8) and fluorescent acceptor (GFP2) across the cell population, where increased interaction correlates with an increased acceptor-to-donor emission ratio. In cells expressing RIβ^WT^, cAMP stimulation decreased RIβ:Cα interactions, whereas minimal change was measured with RIβ^R335W^ :Cα (*p* = 0.000340) (Figure 4A). This reduced R:C dissociation could indicate decreased Cα activation. Therefore, we assayed PKA activity in live cells, taking advantage of our ultrasensitive excitation ratiometric A-kinase activity reporter (ExRai-AKAR2) ^78,79^ co-expressed with mRuby2-tagged RIβ in RIα KO HEK293T. This single-fluorophore biosensor serves as a surrogate PKA substrate and reports PKA activity via fluorescence changes. To observe a more physiologically relevant response, we induced PKA activity in cells expressing either RIβ^WT^ or RIβ^R335W^ using the β-adrenergic receptor agonist isoproterenol (Iso) and calculated total PKA activity as the area underneath the normalized response curves (AUC). Cells overexpressing RIβ^WT^ responded transiently, with a similar temporal profile to previous reports (95% CI of log _10_ mean area = 0.474–1.00),^51^ whereas cells expressing RIβ^R335W^ showed substantially lower Iso-stimulated activity (95% CI[log_10_ mean area] R335W = 0.109–0.235, *p* = 2.26 × 10^−4^ ) (Figure 4B). To extrapolate differences between variants, we also conducted PKA activity assays under maximal cAMP stimulation, which yielded the same trend despite similar basal PKA activity levels between RIβ ^WT^ and RIβ^R335W^ (measured as the decrease in ExRai-AKAR2 excitation ratio following addition of the PKA inhibitor H89) (Figures S4A and S4B).

Because the R335W variant dramatically decreased cAMP-stimulated PKA activity compared with RIβ^WT^, we compared it to the E202A and E326A point mutations, using RIβ^ΔcAMP^ as a control. Iso stimulation failed to induce a PKA response in cells expressing either point mutant (Figure S4C), and only RIβ^E326A^ - expressing cells showed a detectable response to Fsk/IBMX (Figure S4D). RIβ^ΔcAMP^ expression completely blocked PKA activation under both stimulation conditions (Figures S4C and S4D). Thus, despite their ability to undergo LLPS, individual CNB domain mutants fully inhibit PKA signaling in live cells. We then turned our attention to the downstream functional outcomes of the R335W mutation. PKA phosphorylates CREB, a transcription factor that governs long-term potentiation and the consolidation of short- to long-term memory, deficits that are associated with aberrant RIβ expression or variants. ^23,25,26^ Indeed, RIα KO HEK293T cells expressing RIβ^R335W^-mRuby2 demonstrated lower levels of cAMP-stimulated CRE transcription than cells expressing RIβ^WT^ (*p*_Iso_ = 0.00814; *p*_Fsk/IBMX_ = 1.60 × 10^−8^) (Figure 4C). These data, combined with our characterization of RIβ CNB mutant LLPS, support a model of complete disruption of cAMP binding-induced events by R335W, including cAMP-induced LLPS, holoenzyme activation, and downstream signaling. In this case, the C subunit is trapped in the holoenzyme by a cAMP-binding-deficient RIβ mutant, decreasing the pool of activatable C subunit and reducing CREB-mediated transcription.

### The neurodegenerative L50R mutation produces aberrant condensates

**Figure 5.**
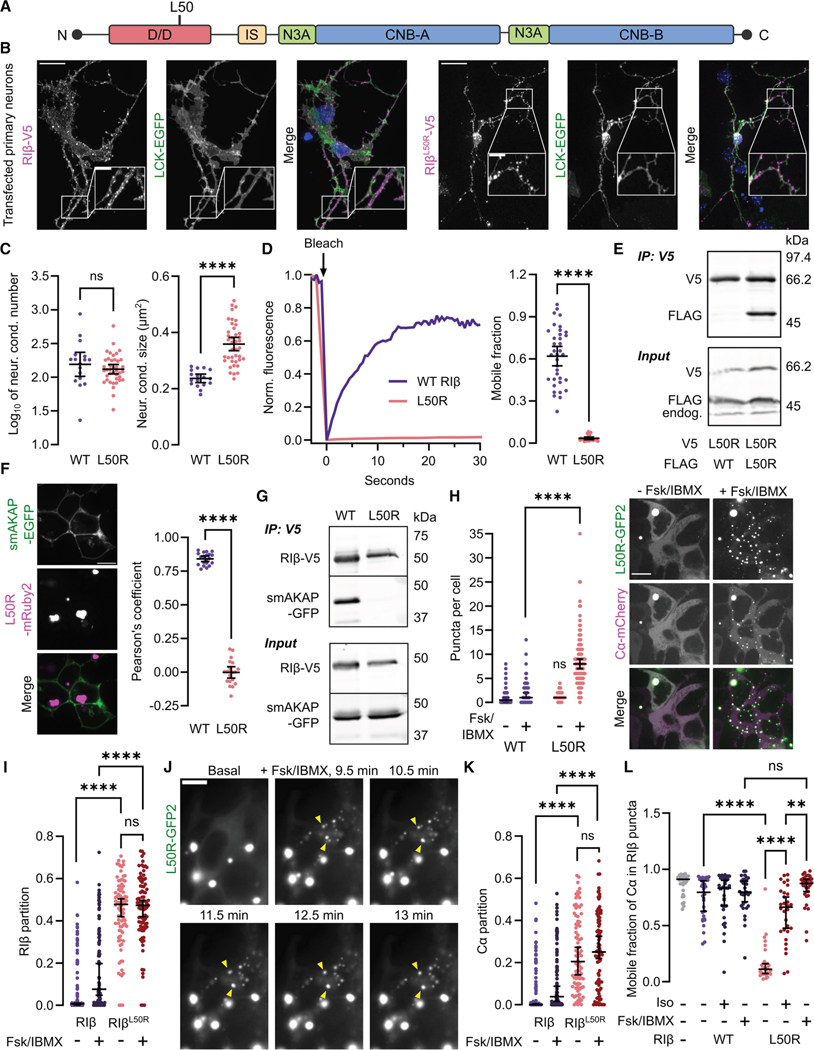
Neurodegenerative L50R disease mutation leads to aberrant condensate formation (A) Domain architecture of RIβ indicating L50. (B) Representative images of RIβ^WT^ - and RIβ^L50R^-V5 assemblies in primary cortical/hippocampal neurons and (C) quantification of the number and average size of assemblies/neuron. *n* = 17 (RIβ^WT^ ), 40 neurons (RIβ^L50R^ ). *p*_number_ = 0.339; ****p_size_ = 2.11 × 10^−8^, unpaired Student’s t test. (D) Representative recovery curve and mobile fractions of photobleached WT- and RIβ^L50R^-GFP2 puncta. *n*_WT_ = 36, *n*_L50R_ = 23 puncta plus 11 with no measurable recovery (DNR) over 5 min. ****p = 1 × 10^−15^, Welch’s t test. (E) Representative co-immunoprecipitation blot showing RIβ^L50R^-V5-LgBiT pulls down RIβ^L50R^-FLAG, but not RIβ^WT^-FLAG. Representative of four independent blots. (F and G) Images, Pearson’s correlations, and co-immunoprecipitation showing smAKAP does not recruit RIβ^L50R^. ****p < 1 × 10^−15^, *n* = 20 whole images. Representative of three independent experiments each. (H) Quantification of puncta per cell and representative images from RIβ^L50R^-GFP2 expressing cells before (−Fsk/IBMX) and after (+Fsk/IBMX) cAMP stimulation. 95% CI_basal_ = 1− 1, 95% CI_cAMP_ = 7−9 puncta, *n* = 114 cells, *p*_WT/L50R basal_ *=* 0.205, ****p_WT/L50R+Fsk/IBMX_ < 1 × 10^−15^, Welch’s t tests. (I) Partition coefficients of RIβ into condensates in cells co-expressing Cα-mCherry. *n*_RIβ_ = 101, *n*_L50R_ = 86 cells, *p*_L50R±Fsk/IBMX_ = 0.0722, ****p_WT/L50R−cAMP_ = 1 × 10^−15^, ****p_WT/L50R+cAMP_ = 1 × 10^−15^, Mann-Whitney unpaired or Wilcoxon paired t tests. (J) Time-lapse images of Fsk/IBMX-stimulated cells co-expressing RIβ^L50R^-GFP2 and Cα-mCherry, showing RIβ^L50R^ puncta fusion. (K) Partition coefficients of Cα in RIβ^L50R^ condensates. *p*_L50R±Fsk/IBMX_ = 0.861, ****p_WT/L50R_ < 1 × 10^−15^, ****p_WT/L50R+Fsk/IBMX_ = 4.31 × 10^−9^, Mann-Whitney unpaired or Wilcoxon paired t tests. *n*_RIβ_ = 101, *n*_L50R_ = 86 cells. (L) Mobile fractions of photobleached Cα-GFP2 in RIβ-mRuby2 condensates with 20 min cAMP stimulation. Recovery of singly expressed Cα-GFP2 shown as a control (gray), *n* = 31 ROIs. For RIβ^WT^, *n* = 33, 34, 31; for RIβ^L50R^, *n* = 28 plus 4 DNR, 30, 32 (left to right). ****p_WT/L50R basal_ < 1 × 10^−15^, ****p_L50R±Iso_ = 1.71 × 10^−9^, **p_L50R Iso/Fsk+IBMX_ = 0.00118, Brown-Forsythe and Welch ANOVA with Dunnett’s multiple comparisons test. (H), (I), (K) and (L), median ± 95% CI; (C), (D), and (F), mean ± 95% CI. All experiments except (B) and (C) were conducted in HEK293T. Some WT data reproduced from Figures 1 and S1, Cα-GFP2 FRAP reproduced from Figure S3. Scale bars, 10 μm; insets, 5 μm. See also Figure S5.

The RIβ L50R mutation was identified as causative in a novel neurodegenerative disorder characterized by parkinsonism with specific aggregates in patient hippocampus, later termed NLPD-PKA. ^31,35^ However, the nature of the L50R “aggregates” and their functional impact were not clear. L50R lies at a critical interface within the D/D domain (Figure 5A), and we recently showed this mutation completely abrogates RIα LLPS by disrupting the dimer interface.^55^ We therefore hypothesized this mutation would greatly alter the properties of RIβ LLPS. Primary neurons from the cortex and hippocampus of neonate mice transfected with RIβ^L50R^-V5 demonstrated large cytosolic assemblies, similar in appearance to those reported in individuals carrying the RIβ^L50R^ mutation (Figure 5B). To mitigate overexpression artifacts in neurons, we used “replacement” plasmids expressing both an RIβ-targeting shRNA and an RNAi-resistant RIβ cDNA from the same backbone. Neuronal L50R condensates were larger than those of RIβ^WT^ but similar in number (95% CI RIβ^WT^ condensate size: 0.221–0.252 vs. RIβ^L50R^ : 0.335–0.382 μm^2^; 95% CI log _10_ (number of RIβ^WT^ condensates): 2.01–2.37, RIβ^L50R^ : 2.05–2.19, *n* = 17, 40 neurons respectively) (Figure 5C). HEK293T cells expressing RIβ^L50R^ - mRuby2 also showed large cytosolic assemblies (Figure S5A). RIβ^L50R^ condensates demonstrated little to no recovery after photobleaching (95% CI_mobile fraction_ = 0.0215–0.0355, *n* = 23 puncta, plus 11 did not recover [DNR]) (Figures 5D, S5B and S5C), suggesting that this disease mutation dramatically affects the biophysical properties of RIβ condensates.

Due to the striking difference in biophysical properties between RIβ^WT^ and RIβ^L50R^ assemblies, we wondered how RIβ^L50R^ condensates behave in the context of RIβ interaction partners, namely RIβ itself, AKAPs, Cα, and cAMP. We first investigated the effect of potential dimerizing partners, RIβ^WT^ and RIβ^L50R^. Co-immunoprecipitation of RIβ^WT^ or RIβ^L50R^ confirmed that each can self-associate, while RIβ ^L50R^ did not associate with RIβ^WT^ (Figures 5E and S5D). We validated this result with both BRET and NanoBiT luciferase complementation assays, which indicated a greater self-association tendency for RIβ ^L50R^, consistent with higher-order homotypic interactions compared with the native dimer (Figures S5E and S5F). Additionally, while RIβ^WT^-EGFP and RIβ^WT^-mRuby2 puncta showed broad colocalization in co-transfected HeLa cells, RIβ^L50R^-mRuby2 formed segregated condensates when co-expressed with RIβ^WT^-EGFP (Figure S5G). These data further differentiate the condensate properties between WT and neurodegenerative mutant RIβ.

Next, we tested if AKAPs could negatively regulate the aberrant L50R assemblies. Cells expressing RIβ^L50R^ still formed cytosolic condensates in the presence of smAKAP, while RIβ^WT^ re-localized to the plasma membrane and condensate formation was inhibited (Figures 1J and 5F), suggesting that RIβ^L50R^ lacks smAKAP binding. We confirmed the loss of RIβ^L50R^ :smAKAP interaction by co-immunoprecipitation and luciferase complementation (Figures 5G and S5H). The effect was also reproduced when we replaced the smAKAP lipidation sequence with a nuclear localization sequence (AKAP_nuc_), which resulted in RIβ ^WT^ recruitment to the nucleus by AKAP_nuc_-BFP2, whereas RIβ^L50R^ localization was unaffected (Figure S5I). Although smAKAP specifically anchors RI isoforms, ^56^ we diversified our approach by probing dual-specific (d)AKAP1, which recruits RI- and RII-containing PKA holoenzymes to the outer mitochondrial membrane, ^80,81^ a process found neuroprotective in an AD model.^82^ Again, RIβ^WT^, but not RIβ^L50R^, co-localized with dAKAP1-EGFP in co-expressing cells, validated by luciferase complementation (Figures S5J–S5L). These data indicate that RIβ ^L50R^ does not bind to AKAPs and is therefore not subject to condensate inhibition by AKAPs, which may in turn enhance RIβ^L50R^ condensate formation and gel-like behavior.

We further investigated the effects of Cα and cAMP on condensate behavior. Co-expressing Cα-mCherry significantly inhibited puncta formation by RIβ^L50R^-GFP2, which showed similar numbers of spherical puncta per cell as RIβ^WT^ in the basal state (*p*_WT/L50R_ *=* 0.205) (Figure 5H). Fsk/IBMX addition dramatically induced RIβ^L50R^ puncta formation (95% CI_basal_ = 1−1, 95% CI_+ cAMP_ = 7−9 puncta per cell, *n* = 114 cells, *p*_WT/L50R_ < 1 × 10^−15^ ) (Figure 5H). We next considered the partitioning behavior of both variants. Although the partitions of RIβ^L50R^ did not increase with Fsk/IBMX treatment (*p* = 0.0722), in both conditions, RIβ^L50R^ partitioning into puncta was more than double that of RIβ^WT^, with between 41.9% and 49.7% of L50R summed intensity correlating to puncta vs. 4.78%–19.8% in cAMP-stimulated RIβ^WT^-expressing cells (95% CI_median_; *p*_+cAMP_ < 1 × 10^−15^ ) (Figure 5I). Interestingly, RIβ^L50R^ assemblies containing co-expressed Cα-mCherry showed slightly greater RIβ^L50R^ recovery upon photobleaching compared with the single-expression case (95% CI_mobile fraction_ [L50R_+Cα_ ] = 0.0297–0.0702, *n* = 26 plus five DNR, *p* = 0.0186, *n*_alone_ = 23, *n*_+Cα_ = 26 condensates) (Figure S5C). Stimulating co-expressing cells with Fsk/IBMX did not further affect the mobile fractions of RIβ^L50R^ in condensates (95% CI_+Cα_ = 0.0324–0.117, *n* = 23 and four DNR, *p* = 0.387), indicating that co-expressed Cα may partly recover RIβ^L50R^ exchange properties (Figure S5C). Again, we observed newly formed puncta fusing during cAMP stimulation, further suggesting that these assemblies retain some liquid-like properties despite their overall gel-like characteristics (Figure 5J). Thus, the presence of Cα could allow some dynamic tuning of RIβ^L50R^ condensate properties.

Like RIβ^WT^, Cα was recruited to RIβ^L50R^ assemblies (Figure 5H). Because the L50R neurodegenerative mutation dramatically altered RIβ condensate properties, we anticipated that co-localized Cα may likewise be affected. Indeed, the basal partition of Cα in RIβ^L50R^ assemblies was dramatically elevated compared with RIβ ^WT^ assemblies (95% CI_L50R_ : 14.2%–27.3% vs. 0.00% ± 0.420%, *p*_basal_ < 1 × 10^−15^ ) (Figure 5K), and Cα exchange with the diffuse pool was dramatically reduced (95% CI_mobile fraction_ = 0.0735–0.160, *n* = 28 and four DNR, *p*_WT/L50R_ < 1.0 × 10^−15^ ) (Figures 5L and S5M), suggesting Cα is trapped in RIβ^L50R^ condensates. We wondered whether the difference in partition and exchange is due to aberrant RIβ^L50R^ phase separation or altered R:C dynamics. To test whether the L50R mutation affects R:C interaction dynamics, we conducted R:C BRET experiments and found no defective RIβ^L50R^ :Cα holoenzyme dissociation or re-association (Figure S5N). Trapping of Cα also occurs in the presence of high cAMP. Despite regaining exchange with the cytosol (mobile fraction *p*Cα±Iso = 1.71 × 10^−9^, *p*WT/L50R+Fsk/IBMX = 0.961), Cα maintained its higher partition into RIβ ^L50R^ condensates after cAMP stimulation (*p*_L50R±Fsk/IBMX_ = 0.861) (Figures 5K, 5L, and S5M), indicating Cα is retained in gel-like RIβ^L50R^ assemblies despite high cAMP levels. Together, the data indicate that the RIβ^L50R^ mutation induces the formation of aberrant, gel-like condensates that sequester a large proportion of PKA C (here, Cα) rather than affecting the R:C holoenzyme.

### L50R impairs PKA signaling

**Figure 6.**
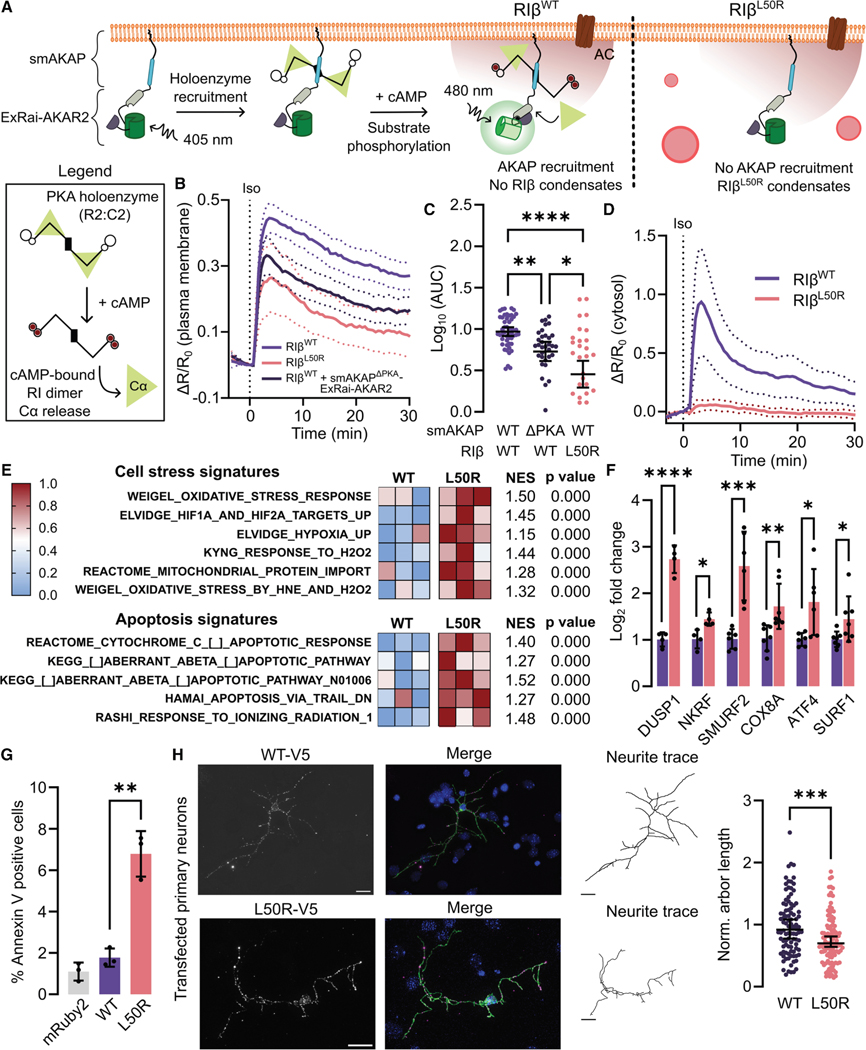
Dementia-linked L50R mutation is associated with cell death and reduced neurite outgrowth (A) Graphic illustrating assay to measure smAKAP-proximal PKA activity, using smAKAP ^ΔPKA^ as a control. (B and C) PKA activity curves and log _10_ transform of total activity (area under the curve). **p_smAKAP/ΔPKA_ = 0.00130, ****p_WT/L50R_ = 7.38 × 10^−7^, *p_ΔPKA/L50R_ = 0.0193 by Dunnett’s T3 multiple comparison test; *n*_WT_ = 50, *n*_L50R_ = 36, *n*_ΔPKA_ = 34 cells. (D) Cells expressing RIβ^L50R^ show substantial deficits in cytosolic PKA signal in response to 5 μM Iso. *n*_WT_ = 23, *n*_L50R_ = 18 cells; WT data reproduced from Figure 4. (E) Heat maps showing representative apoptosis- and cell stress-related gene set enrichment profiles. (F) RT-PCR validation of differentially expressed genes. ****p_*DUSP1*_ = 4.84 × 10^−5^, *p_*NKRF*_ = 0.0135, ***p_*SMURF2*_ = 5.35 × 10^−4^, **p_*COX8A*_ = 0.00707, *p_*ATF4*_ = 0.0215, *p_*SURF1*_ = 0.0459, unpaired t tests. (G) Percentage of RIβ, RIβ^L50R^, or mRuby2-expressing cells staining for Annexin V after 48 h transfection, measured by flow cytometry. **p = 0.00182, unpaired t test. (H) Measurements of dendritic arbors of mixed primary hippocampal and cortical neurons from neonate mice co-transfected with RIβ or RIβ^L50R^ and LCK-GFP, normalized to the mean wild-type arbor length of each independent experiment. Scale bars, 15 μm; *n* = 96 (RIβ^WT^ ), 115 neurons (RIβ^L50R^ ), ***p = 0.000640, Mann-Whitney test. Experiments in (A)–(G) conducted in RIα KO HEK293T. Plots are mean ± 95% CI (B)–(D), mean ± SD (F) and (G), or median ± 95% CI (H). All data are from at least three independent experiments. See also Figure S6.

We reasoned that impaired AKAP binding to RIβ^L50R^ and Cα retention in RIβ^L50R^ condensates may lead to dysregulated PKA signaling at AKAP-assembled microdomains. To measure this effect, we tethered ExRai-AKAR2 to smAKAP to compare smAKAP-proximal PKA activity between RIβ^WT^ or RIβ^L50R^ (Figure 6A). As expected, we observed greater Iso-stimulated plasma membrane PKA responses in cells co-expressing RIβ^WT^ with smAKAP-ExRai-AKAR2 than with smAKAP^ΔPKA^ -ExRai-AKAR2 (95% CI log_10_ (AUC) = 0.915–1.02, *n*_smAKAP_ = 50, smAKAP^ΔPKA^ : 0.614–0.846, *n*_ΔPKA_ = 34 cells, *p* = 0.00130) or cells co-expressing RIβ^L50R^ (95% CI = 0.293–0.616, *n* = 36 cells, *p* = 7.38 × 10^−7^ ). Further, smAKAP-ExRai-AKAR2 and RIβ^L50R^ co-expressing cells responded even less than cells co-expressing RIβ ^WT^ and the ΔPKA sensor (*p* = 0.0193) (Figures 6B and 6C). This difference extended to unstimulated PKA activity at the smAKAP microdomain, where cells expressing RIβ^L50R^ exhibited the lowest basal PKA activity (Figure S6A). These data indicate that L50R leads to a loss of AKAP-mediated PKA signaling microdomains, decreasing AKAP-directed local PKA activity.

The strong retention of Cα in RIβ^L50R^ condensates further prompted us to test for global PKA activity deficits. Strikingly, Iso elicited minimal cytosolic PKA activity, with an almost 8-fold lower response in RIα KO cells expressing RIβ^L50R^ compared with RIβ^WT^ (RIβ^WT^ : 95% CI log_10_ mean area = 0.474–1.00, *n*_WT_ = 23 cells, RIβ ^L50R^ : − 0.103−0.202, *n*_L50R_ = 18 cells, *p* = 4.20 × 10^−5^) (Figures 6D and S6B). This difference extended to but was less dramatic upon Fsk/IBMX stimulation, where cells expressing RIβ ^L50R^ showed lower response amplitudes than RIβ^WT^ -expressing cells (Figure S6C), suggesting supraphysiological stimulation could only partially overcome the signaling deficit. Altogether, our data support a model in which both local and global PKA activity is dysregulated by RIβ^L50R^, with almost complete suppression of cytosolic PKA activity concomitant with sequestration of Cα in aberrant condensates.

### RIβ^L50R^ induces hallmarks of neurodegeneration

To bridge the L50R-induced PKA activity defects and functional outcomes, we conducted RNA-seq of RIα KO cells overexpressing RIβ^L50R^, RIβ^WT^, and mRuby2, as a control. We evaluated the transcriptome of L50R-expressing cells both on the level of individual genes (Figure S6D; Table S1) and by gene ontology using gene set enrichment analysis (GSEA) (Table S2). Strikingly, we observed substantial enrichment for signatures related to cell stress and apoptosis in the L50R condition (Figure 6E; Table S2). The GSEA output was echoed at the differentially expressed gene (DEG) level by significant upregulation of apoptosis- and stress response-related genes, including *DUSP1*,^83,84^ *SMURF2*,^85,86^ and *ATF4*,^87,88^ which we validated by RT-PCR (Figure 6F). We were particularly interested in the enrichment of apoptosis pathways because people with NLPD-PKA exhibit accelerated neuronal cell loss.^31^ To corroborate the transcriptional profile of RIβ^L50R^ with its phenotypic outcome, we used flow cytometry to measure the proportion of Annexin V-stained RIα KO HEK293T cells overexpressing RIβ^WT^, L50R, or mRuby2 control. Cells with high RIβ expression exhibited 4-fold higher phosphatidylserine-positive staining in the L50R condition 48 h post-transfection compared with RIβ^WT^ (mean percent Annexin V-positive RIβ^WT^ cells = 1.77% ± 0.25%, RIβ^L50R^ = 6.79% ± 0.63%, *p* = 0.00182) (Figure 6G), suggesting increased apoptosis for cells containing RIβ^L50R^.

Finally, we sought to validate the RIβ^L50R^ -induced effects in a neuronal system. Recent work suggests that molecular defects associated with neurodegenerative diseases also influence brain structure and function during an extended period of brain development.^89–91^ As PKA signaling is a critical regulator of neuritogenesis,^92–95^ we hypothesized that the decreased PKA signaling induced by RIβ^L50R^ would lead to decreased neurite outgrowth in developing neurons. To test this hypothesis, we isolated primary neurons and performed gene replacement using small hairpin (sh)RNA targeted against endogenous RIβ and re-expression of RNAi-resistant RIβ^WT^ or RIβ^L50R^ to investigate the lengths of dendritic arbors of individual neurons. As shown in Figure 6H, we found that neurite outgrowth was significantly decreased in cells expressing RIβ^L50R^ (*p* = 0.000640), highlighting an overall functional deficit induced by this neurodegenerative variant. The data collectively lead to a model wherein cells containing aberrant RIβ^L50R^ biomolecular condensates demonstrate a loss of compartmentalized and global PKA signaling, leading to functional deficits including elevated apoptosis and decreased neurite outgrowth. These findings not only contribute to the mechanistic understanding of NLPD-PKA but also illustrate the critical role of physiological RIβ condensates in maintaining neuronal function.

## DISCUSSION

**Figure 7.**
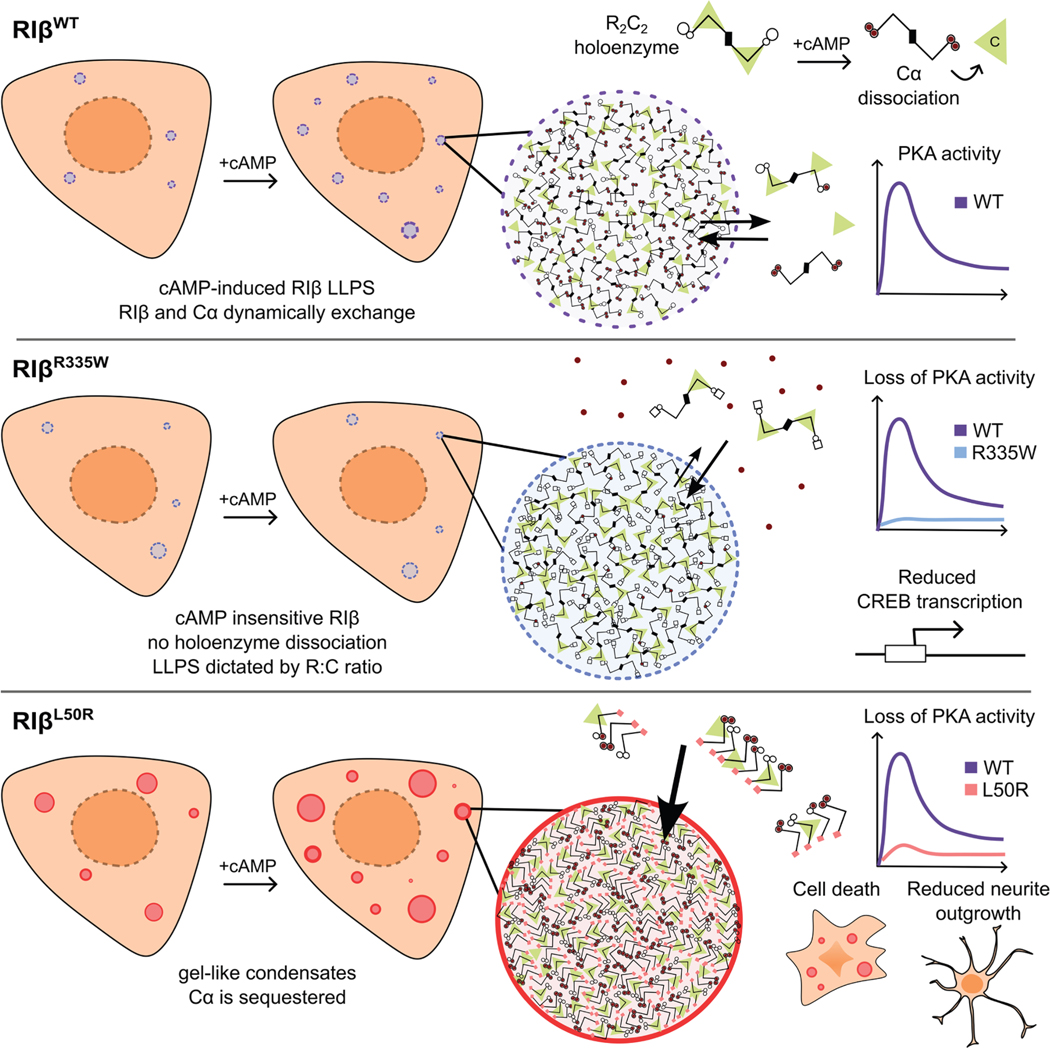
Graphical summary of physiological PKA RIβ LLPS compared to aberrant phase separation in two distinct neurological diseases leading to dysregulated PKA signaling and other functional outcomes Graphic indicates relative changes in condensate properties, response to cAMP stimulation, and functional consequences leading to the R335W-driven MASNS and L50R-driven NLPD-PKA disease states, respectively.

Our work supports a model of RIβ phase separation in which PKA exists in equilibrium between the diffuse, inactive holoenzyme and RI-driven condensates that contain Cα, both of which dynamically exchange with the cytosol, mediated by cAMP dynamics (Figure 7). However, RIβ has distinct conformational space from RIα, making it not only prone to condensate formation but also susceptible to assembly into condensates of diverse material properties. A critical molecular determinant of RIβ is the intrinsically disordered linker. In the inactive holoenzyme, this linker is buried near the Cα active site cleft (Figure 2C); cAMP-induced RIβ LLPS involves cAMP binding to both CNB domains and subsequent conformational changes that allosterically unleash the C subunit, increasing exposure and subsequent disordering of the linker. The disorder-promoting or spacer-like characteristics of the linker not only promote phase separation but provide conformational flexibility to prevent RIβ forming higher-order fibers. Intriguingly, the presence of Cα recovers liquid-like condensate formation in RIβ^ΔLinker^ (Figures 2D and 2E), highlighting a more active role for PKA C in RI condensate formation than previously appreciated, especially for facilitating the conformational pathway to liquid-like condensates.

R335W in MASNS and L50R in NLPD-PKA are mutations that cause distinctly aberrant phase separation of RIβ and contrasting mechanisms of C subunit sequestration. Although RIβ^R335W^ is fully capable of undergoing LLPS, its cAMP insensitivity re- moves it from the holoenzyme-to-condensate equilibrium (Figure 3E). This leads to partitioning, which is dependent on the relative cellular concentrations of R and C—independent of physiological cAMP fluctuations. Thus, PKA C is permanently sequestered within inactive holoenzymes, suppressing PKA activity (Figure 7). As RIβ is highly expressed during embryonic development ^96^ and underlies pain sensitivity, ^23^ LTP, ^25^ and synaptic plasticity, ^19^ this mutation is well-poised to impede neuronal development via global PKA inhibition and may explain the neurodevelopmental phenotypes and pain insensitivity observed in people with MASNS.

While RIβ^R335W^ sequesters PKA C in the holoenzyme, PKA C sequestration by RIβ^L50R^ is mediated by its aberrant condensate properties. In several neurodegenerative diseases, liquid-to-gel or liquid-to-aggregate transitions by intrinsically disordered proteins are a proposed mechanistic explanation for disease pathology, leading to aberrant proteostasis, stress, and cell death. ^97,98^ Here, we show that RIβ, a protein generally considered as ordered, is capable of transitioning from physiological liquid-like assemblies to pathological gel-like assemblies due to a point mutation. Mechanistically, loss of the well-defined dimer interface likely triggers specific conformational transformations of RIβ ^L50R^ that are capable of nucleating stable self-interactions to drive unique assembly pathways, arriving at gel-like condensates (Figures 5D, 5E, and 7). Lack of AKAP binding likely further enhances the formation of gel-like condensates (Figures 5F and 5G). Despite their gel-like properties, RIβ^L50R^ assemblies bear many similarities to their liquid-like wild-type counterparts vs. traditional insoluble, irregular aggregates and inclusion bodies. For example, the formation of L50R assemblies can, to a degree, be regulated by cAMP and Cα in the same fashion as WT, further highlighting the active role of Cα in driving the conformational pathway to liquid-like RI condensates (Figure 5H). In addition, Cα is also recruited to L50R condensates, as with RIβ^WT^, although cytosolic exchange is limited by the gel-like properties, leading to Cα trapping (Figures 5K and 5L). Altogether, our data support a model of NLPD-PKA in which RIβ^L50R^ forms highly partitioned gel-like condensates that can retain substantially more Cα than RIβ^WT^ condensates (Figures 5I, 5K, and 5L), dysregulating PKA activity both globally and at AKAP signaling microdomains, leading to several hallmarks of neurodegenerative disease including apoptotic cell death and decreased neurite outgrowth (Figure 6). PKA C trapping by aberrant RIβ^L50R^ condensates provides a striking mechanistic example of how aberrant molecular assemblies lead to defective signaling and dysregulated functional activity.

Based on this work and the clinical phenotypes seen in individuals with MASNS and NLPD-PKA, we suggest that the monoallelic R335W and L50R variants induce aberrant phase separation behaviors that cripple PKA C in distinct manners: one locking it within the holoenzyme and the other trapping it in gel-like condensates. Our findings are consistent with the conclusion that RIβ^R335W^ causes MASNS by acting as a dominant-negative mutant, whereas RIβ^L50R^ leads to NLPD-PKA via *PRKAR1B* loss-of-function or haploinsufficiency. This is supported by initial reports of RIβ^R335W^ MASNS onset occurring during gestation or infancy,^26,27^ whereas NLPD-PKA strikes much later in life, at 45–64 years of age.^31^ This study provides a stark example of how changes in the biophysical properties of macromolecules can tip physiological signal tuning into pathological molecular trapping, in this case ultimately translating to neurological disease. By contrast, although both RIβ^R335W^ and RIβ^L50R^ strongly diminish PKA activity to arrive at their disease states, our previous work implicated loss of PKA RIα condensates in enhanced cell proliferation and transformation in fibrolamellar carcinoma^51^; thus, aberrant condensate formation may lead to too much or too little signaling, causing cancer or neurological disease, respectively. As we continue to investigate how the intrinsic phase separation tendency and versatility of different macromolecules are exploited and amplified in dis- ease settings, investigations will certainly benefit from characterization of both molecular assemblies and the resulting functional activities in living cells, providing opportunities for new therapeutic approaches targeting these devastating and still incurable diseases.

### Limitations of the study

Our work provides evidence for PKA RIβ LLPS and reveals aberrant phase separation behaviors of two distinct neurological dis- ease mutants. While the experimental approaches used in this study allowed us to probe the biophysical and molecular properties of RIβ condensates, as well as their functional impacts, technical limitations preclude us from fully recapitulating the endogenous neuronal environment. For example, gene replacement studies only approximate native conditions and cannot ensure physiological RIβ expression levels. Genetically modified mouse models will be crucial for obtaining further insights into the contribution of disrupted RIβ LLPS to disease initiation and progression *in vivo*.

## RESOURCE AVAILABILITY

### Lead contact

Inquiries and requests for resources or reagents should be directed to the lead contact, Jin Zhang (jzhang32@health.ucsd.edu).

### Materials availability

Plasmids generated in this study will be made available through Addgene.

### Data and code availability

- Data reported in this paper will be shared by the lead contact upon request.
- RNA-seq data have been deposited to the NCBI Gene Expression Omnibus (GEO) platform and are publicly available as of the date of publication. Accession numbers are listed in the key resources table.
- This paper analyzes existing, publicly available data, accessible at the RCSB Protein DataBank with PDB: 4DIN, 4MX3.
- This paper does not report original code.
- Any additional information required to reanalyze the data reported in this paper is available from the lead contact upon request.

## STAR★METHODS

### EXPERIMENTAL MODELS

#### Cell culture and transfection

HEK293T, RIα KO HEK293T, and HeLa cells were cultured in Dulbecco’s modified Eagle medium (DMEM, GIBCO) containing 4.5 g L^−1^ (293T) or 1 g L^−1^ (HeLa) glucose, supplemented with 10% (v/v) fetal bovine serum (FBS, Sigma), and 1% (v/v) penicillin-streptomycin (Pen-Strep, Sigma). COS-1 cells were cultured as HEK293T cells with added 1x GlutaMax (GIBCO). Cells were grown in a 37°C incubator with a humidified 5% CO_2_ atmosphere. Cells were plated on sterile poly-lysine-coated 35-mm glass-bottomed dishes, glass coverslips, or poly-lysine- or 1% collagen-coated 96-well plates (Corning) and grown to 50–70% confluence before transient transfection using PolyJet (Signagen) and grown for a further 16–48 h (293T) or with Lipofectamine 2000 (Invitrogen) and grown an additional 16–24 (COS-1) or 16–48 h (HeLa) as indicated. PC12 cells were cultured in HEK293T DMEM additionally supplemented with 5% donor horse serum (GIBCO) and cultured for 48 h after transfection with Lipofectamine 2000 (Invitrogen).

#### Mice

Mouse work was performed in accordance with the guidelines of the animal ethics committee of the University of Iowa. Mice were group-housed in a colony maintained with a standard 12 h light/dark cycle and given food and water *ad libitum*. Euthanasia prior to neuronal culturing was conducted according to the Guide for the Care and Use of Laboratory Animals, as adopted by the National Institutes of Health, and with approval of the University of Iowa AAALAC-accredited Institutional Animal Care and Use Committee.

#### Primary neuronal cultures

For each independent neuronal culture experiment, primary mixed hippocampal and cortical neuronal cultures were prepared by isolating cortices and hippocampi from 8 to 16 postnatal day 0 to postnatal day 1 (P0-P1) wild-type (WT) C57BL/6J male and female mice. Cortices and hippocampi were dissected in ice-cold HBSS, pooled in ice-cold Neurobasal Plus (NB+) medium (GIBCO), and incubated in HBSS containing 1.5 mg/mL trypsin at 37°C for 15 min. Afterward, the trypsin solution was removed and neutralized via NB + complete medium with 5% heat-inactivated horse serum (NB+ complete +5% HI-HS; NB + medium supplemented with 2% B27 Plus Supplement (GIBCO), 10 mM HEPES (GIBCO), 0.25% GlutaMax (GIBCO), and 5% heat-inactivated horse serum (HI-HS)). Brain tissue was rinsed twice in HBSS and dissociated by trituration in HBSS. After filtration (40 μm filter, Fisherbrand), cells were pooled together, regardless of the sex of each individual mouse. Next, cells were plated in NB+ complete +5% HI-HS on poly-lysine- and laminin-coated glass-bottom 24-well plates at 1.2 × 10^5^ cells/well. After 4 h, the medium was replaced with NB+ complete +5% HI-HS medium without heat-inactivated horse serum. Cells were maintained at 37°C with a humidified 5% CO_2_ atmosphere.

#### Neuronal transfections

DIV2 primary neuronal cultures were transfected using NeuroMag transfection reagent (OZ Biosciences) with a super-magnetic plate (OZ Biosciences) according to the manufacturer’s instructions two days after seeding in 24-well plates. Neuronal cultures were transfected using a NeuroMag ratio of 2 (NeuroMag Ratio = Volume of NeuroMag Reagent (in μL)/Amount of DNA Plasmid (in μg)) and a total of 0.6 μg DNA/well. Briefly, NeuroMag transfection reagent in NB + medium was added to microcentrifuge tubes containing DNA constructs at a ratio of 75% V5-His6-tagged RIβ replacement plasmid (see below) and 25% LCK-GFP, after which 150 μL transfection mixture was immediately added per well. The 24-well plate was placed on the magnetic plate and incubated at 37°C, 5% CO_2_ for 20 min, followed by removal of the magnetic plate and incubation for another 36–48 h.

### METHOD DETAILS

#### Plasmid construction

Constructs were generated in the pcDNA3.1 (Invitrogen), pCMV6 V5-His_6_ (Origene), or pEGFP-N1 (Takara) backbones, unless specified otherwise, and verified by Sanger sequencing (Genewiz or U. Iowa Genomics Division). Primers used to generate plasmids are listed in Table S3.

##### Tagged RIβ constructs

RIβ-GFP2 was generated via PCR amplification of GFP2 from pcDNA3-GFP2-RIα and human RIβ cDNA, followed by Gibson assembly using the NEBuilder Hi-Fi DNA Assembly Cloning Kit (New England Biolabs). Domain deletion and point mutants were generated by Gibson assembly, amplified from RIβ-GFP2. RIβ-mRuby2 and point mutants were generated by Gibson assembly using RIβ-GFP2 or variants and RIα-mRuby2 as templates. RIβ^R335W^ -mTagBFP2 was generated by Gibson assembly by replacing GFP2 with mTagBFP2 from mTagBFP2-RIα. ^51^

RIβ-V5 was generated using Gibson assembly, amplified from RIβ-GFP2; the V5 tag was added via primer drop-in. pEGFP-N1 RIβ-FLAG WT and mutants were generated by PCR-amplification of WT and mutant RIβ and insertion of the amplicon into the *Sal*I and *Hind*III sites of pEGFP-N1. pCMV6 LgBiT/SmBiT-RIβ-V5 was constructed in two steps. First, WT and mutant RIβ PCR-amplicons were inserted into the *Hind*III and *Not*I sites of pCMV6 V5-His_6_. Second, LgBiT and SmBiT, including C-terminal poly-Gly-Ser spacers, were amplified from pNB MCS-3 and pNB MCS-4 (Promega) and inserted into *Nhe*I and *Hind*III sites of pCMV6 RIβ-V5-His_6_. smAKAP-LgBiT was generated by replacing the EGFP coding sequence of pEGFP-N1 smAKAP-FLAG with poly-(Gly-Ser)-LgBiT amplified from pNB MCS-1 (Promega) using *Sal*I and *Not*I sites.

His-hRIβ, used for protein expression, was generated by restriction cloning. ^99^ The coding sequence for PKA-hRIβ was PCR-amplified from an existing pRSETB construct while simultaneously adding *Sal*I and *Hind*III restriction sites, then inserted into a linearized pQTEV vector. Ligation success was evaluated by colony PCR, control restriction digestion, and sequencing.

##### RIβ replacement plasmids

To minimize RIβ overexpression artifacts in neuronal experiments, we generated constructs which simultaneously knock down endogenous RIβ expression with a small hairpin (sh)RNA and overexpress RNAi-resistant RIβ WT or L50R. We selected 6 potential shRNA targets (19 bp) that are complementary to the coding regions of mouse and human RIβ.^108,109^ Oligonucleotides encoding the shRNAs and a common primer annealing to the 5′ end of the H1 promoter were used to amplify H1-shRNA cassettes, which were then ligated into *Bgl*II-digested pcDNA3.1. shRNA potency was assessed by co-transfecting HEK293 cells with serial dilutions of shRNA plasmids together with fixed amounts of firefly luciferase-RIβ fusion protein expression and SV40-driven Renilla luciferase expression plasmids, followed by dual-luciferase assays. The RIβ-V5-His_6_ coding sequence was ligated into the best shRNA expression plasmid (#5) and rendered RNAi-resistant by introducing 4 silent mutations into the shRNA target site.

##### smAKAP-tethered PKA activity biosensor

smAKAP-ExRai-AKAR2 and smAKAP(L65P/L70P)-ExRai-AKAR2 were generated by Gibson assembly using fragments amplified from pcDNA 3.1(+) ExRai-AKAR2, pEGFP-N1 smAKAP-FLAG and pEGFP-N1 smAKAP(L65P/L70P)-FLAG. ^55^

#### Immunofluorescence

PC12 or HEK293T cells were seeded on glass coverslips and allowed to reach 80% confluence with or without transfection, as indicated. Some PC12 cells were stimulated with 50 μM forskolin and 100 μM 3-isobutyl-1-methylxanthine for 30 min at 37°C. Coverslips were washed 2x (PC12) or 3x (HEK293T) with phosphate buffered saline (PBS) and fixed with 4% paraformaldehyde (PFA) in PBS. Coverslips were washed with PBS, permeabilized with PBS containing 0.1% Triton X-100, and blocked in permeabilization buffer containing 1% bovine serum albumin at room temperature for 6 h or overnight at 4° C. Blocked coverslips were incubated with primary antibody (1:300 sheep anti-RIβ for PC12 and 1:1000 mouse anti-V5 for HEK293T) in blocking buffer overnight at 4°C, washed with PBS, and incubated in blocking buffer with secondary antibody (1:2000) for 1 h at room temperature. Labeled coverslips were washed and mounted on glass slides with mounting agent containing NucBlue (Invitrogen) and dried before imaging. HeLa cells were seeded on chambered No. 1 cover glasses (Nalgene Nunc) with or without transfection at 60% confluence. Cells were processed for optional immunofluorescence as described. ^108^ HeLa cells expressing BFP2 fusion constructs were counterstained with RedDot2 far- red nuclear stain (Biotium, 1:200); otherwise, DNA was labeled with Hoechst 33342 (1 μg/mL). Images are representative of three independent experiments.

#### Fixation and staining of primary neuronal cultures

Neuronal cultures were fixed 36–48 h post-transfection with 4% PFA in 1X DPBS for 15 min with incubation at 37°C and a 5% CO_2_ atmosphere. Fixed cultures were washed four times with 1X DPBS for 5 min each, then rinsed once with (5 min) and maintained in 1X DPBS. Fixed neuronal cultures were blocked by incubation in blocking solution (0.5 μM EDTA and 2% normal goat serum in TTBS; TTBS: 154 mM NaCl, 10 mM Tris pH 7.5, 0.1% Triton X-100) for 1 h at room temperature, then stained by incubating with 1:500 mouse anti-V5 primary antibody (Invitrogen) and 1:1000-diluted rabbit anti-GFP primary antibody (Abcam) in blocking solution overnight at 4°C. After primary antibody incubation, cultures were washed four times in TTBS (5 min each), once in DPBS (5 min), and incubated with 1:250 goat anti-rabbit Alexa 488 (Invitrogen) and 1:250 goat anti-mouse Alexa 555 (Invitrogen) antibodies in blocking solution with Hoechst 33342 (1 μg/mL) for 2 h at room temperature with rocking in the dark. Following secondary antibody incubation, neuronal cultures were washed with TTBS and DPBS as above, then maintained in 1X DPBS at 4°C until imaging.

#### Fluorescence microscopy

Cells were washed twice with and imaged in HBSS (GIBCO) at room temperature in the dark. Stimulation with 50 μM Forskolin (Fsk; Calbiochem), 100 μM 3-isobutyl-1-methylxanthine (IBMX; Sigma), 5 μM isoproterenol (Iso; Sigma Aldrich), and 20 μM H89 was performed as indicated.

##### Widefield epifluorescence microscopy

Epifluorescence imaging, including time-lapse, puncta formation, colocalization, cAMP response, and kinase activity assays, was performed on a Zeiss AxioObserver Z1 microscope (Carl Zeiss) equipped with a 40×/1.3 NA objective, Definite Focus (Carl Zeiss), a Lambda 10–2 filter-changer (Sutter Instruments), and a Photometrics Evolve 512 EMCCD camera (Photometrics) controlled by METAFLUOR 7.7 software (Molecular Devices). Excitation and emission filter combinations used were: BFP – EX380/10, EM475/25, GFP - EM 480/30, EX535/45, RFP – EX568/55, EM653/95. Exposure times for each channel ranged from 50 to 500 ms, with electron multiplying gain set between 10 and 50 and identical acquisition parameters between experimental replicates. Time-lapse images were acquired every 30 s. Additional epifluorescence imaging was conducted using a Leica 4000B with LAS X software (Leica), a 100X/1.4 NA oil-immersion objective, and the following filter cubes: DAPI (Ex: 390/70, DC: 455, EM: LP 470); GFP (Ex: 470/80, DC:500, Em: 525/100), CY3 (Ex: 545/26, DC: 565, Em: 605/70), CY5 (Ex: 620/120, DC: 660, Em:700/150).

##### Spinning-disk confocal microscopy

Confocal imaging, including representative images, immunofluorescence imaging, Z-stacks, and fluorescence recovery after photo bleaching, was performed using a Nikon Ti2 microscope equipped with a Yokogawa W1 confocal scan head, Opti Microscan FRAP unit, and operated with NIS Elements software (Nikon) using the 405, 488, 515, and 561 nm lines of a six-line (405, 445, 488, 515, 561, and 640 nm) LUN-F-XL laser engine or brightfield imaging, using an Apo TIRF 100×/1.49 NA objective. Images were re- corded using a Prime95B camera (Photometrics). Z-stacks were acquired using a 0.1- or 0.2-μm step size and 3D reconstructions were generated in FIJI. Image acquisition laser power ranged from 1 to 35% for different applications with 200 ms exposure time, except for DIC, which was performed at 100% LED power and 500 ms exposure. Replicate experiments were acquired with identical acquisition parameters. Where indicated, cells were stained with Hoechst 33342 (1 μg/mL) for 30–60 min prior to washing and imaging.

##### Imaging of neuronal cultures

Fluorescence imaging of primary neuronal cultures was performed on a Keyence BZ-X810 microscope with the PlanApo_λ 100xH 1.45/0.13 mm oil objective using the monochrome 12-bit normal capture mode on the BZ-X800 Viewer software (Keyence). Images of neurons were taken as Z-stacks using approximately 20 focal planes at 1-μm intervals, and acquisition was done under the “High Sensitivity” resolution setting to generate images of 640 × 480 pixels. Images were acquired without digital zoom magnification, white balance, or contrast correction with the following excitation and emission filter parameters: (EX/EM) Cy3: 545/605; GFP 470/525; and DAPI 360/450 nm. All images were taken using the “Sectioning” function with the “1D Slit” set to a width of 10, with the “Brighter” option selected. The following image filters were used for the DAPI channel only: Edge/Emboss – Edge (weak) and Haze Reduction – Setting 1. Images were acquired with exposure times for each channel ranging from 1 to 250 ms using the 2×2 binning mode with the gain set to 6 dB for all channels. Neurons with neurites extending beyond the field of view of the microscope were imaged by specifying the area encompassing each neuron using the “Set Edge Points” option under the “Stitching” function. After image acquisition, images were stitched together using the “Full Focus” and “Sectioning Image” procedures in the BZ-X800 Analyzer software (Keyence). The BZ-X800 Analyzer software was also used to generate images overlaying all 3 channels for each neuron. Images used for display were subjected to contrast enhancement (ImageJ’s “Enhance Contrast …” function) to improve clarity.

#### Fluorescence recovery after photo bleaching

Fluorescence recovery after photobleaching (FRAP) data were acquired using the spinning-disk confocal microscope as described above. Circular regions of interest were drawn over whole puncta or regions of bulk cytosol for photobleaching. RIβ-GFP2: 5–10 pre-bleach images were acquired at 0.5-s intervals using the 488 nm laser line at 12% power, then puncta or bulk cytosolic regions of interest were bleached using the 405 nm laser line for 500 ms at 75% power and recovery monitored every 0.5 s for a total of 2 min. RIβ^L50R^-GFP2 images were acquired every 1 s for a total of 5–10 min. RIβ^ΔLinker^ -GFP2 images were acquired every 0.5 s for 2 min for puncta and every 1 s for 10 min for fibrillar structures. After verifying co-localization where applicable, Cα-GFP2 images were acquired every 0.5–1 s for 2− 5 min with respect to the co-expressed RIβ LLPS system.

#### Protein expression and purification

Human RIβ protein was transformed into and expressed in TP2000 *E. coli*, in which the adenylyl cyclase gene is deleted. ^110,111^ A pre- culture was prepared by inoculating 100 mL LB medium with a single transfected colony and incubated overnight at 37°C with shaking at 135 rpm. 15 mL pre-culture was used to inoculate full liters of cell culture, with incubation at 37°C and shaking at 135 rpm until the culture reached an optical density OD_600_ = 0.6. Protein expression was induced by addition of 400 μM isopropyl β-D-1-thiogalactopyranoside and incubated overnight at 19°C with shaking. Cells were harvested by centrifugation at 9,000 × *g* for 30 min at 4°C and cell pellets stored at − 20°C.

His-tagged RIβ was purified using Ni^2+^ -NTA affinity chromatography (Macherey-Nagel). Cell pellets were resuspended in 5 mL lysis buffer/g pellet (50 mM KH_2_ PO_4_, pH = 8.0, 500 mM NaCl, 20 mM imidazole, 5 mM β-mercaptoethanol (βME), 0.1% Triton X-100) with added protease inhibitor (Sigma Aldrich). Cells were lysed via French Pressure Cell (Thermo IEC French Press, ThermoFisher). Cell lysates were cleared by centrifugation at 45,000 × *g* for 30 min at 4°C and the supernatant incubated with pre-equilibrated Ni^2+^ -NTA agarose for 2 h at 4°C under continuous rotation. Loaded agarose was collected via centrifugation for 2 min at 1,000 × *g*, washed twice with wash buffer containing a low concentration of imidazole (50 mM KH_2_ PO_4_, pH = 8.0, 500 mM NaCl, 60 mM imidazole, 5 mM βME), once with moderate imidazole (wash buffer with 100 mM imidazole), then transferred to a Pierce column for elution with high imidazole elution buffer (wash buffer with 250 mM imidazole). Eluted protein fractions were exchanged to storage buffer (20 mM MOPS, pH = 7.4, 150 mM NaCl, 2 mM EGTA, 2 mM EDTA, 5 mM βME) using a PD-10 desalting column (Cytiva) and frozen at − 20°C. Protein purity was assessed using SDS-PAGE on 4–15% mini-PROTEAN TGX protein gels (Bio-Rad).

#### *In vitro* liquid droplet assays

Purified RIβ protein was dialyzed twice into 0.2 μm filter-sterilized liquid droplet assay buffer (LDB: 20 mM HEPES, pH 7.0, 150 mM KCl, 5 mM MgCl_2_, 1 mM EGTA, 0.5 mM ATP, 5 mM βME), concentrated, and assays completed as previously described ^51,55^ using a final concentration of 20 mg/mL polyethylene glycol 4000 diluted in LDB where indicated. Briefly, purified protein was incubated in glass-bottomed 96-well plates at different concentrations, incubated at room temperature for 30 min-1 h, and imaged using bright- field or differential interference contrast (DIC) imaging at 20x or 100x, respectively. Conditions were determined to have droplets by the presence of defined, clearly visible spherical or elliptical-shaped assemblies at 20x or 100x. Droplets varied in shape and size depending on the relative concentration of the protein solution. Images are representative of 3 experiments.

#### Bioluminescence resonance energy transfer (BRET) assays

RIα KO HEK293T cells were transfected using PolyJet in 6-well plates (Costar) for 24 h, then seeded in poly-lysine coated white-walled and clear-bottom 96-well assay plates (Costar) at a density of 5 × 10^4^ cells per well. Cells were washed twice with HBSS and covered in 100 μL of fresh HBSS containing 5 μM CTZ400a (DeepBlueC, Nanolight Technology) immediately prior to recording, and baseline reading acquired for 8 cycles. To monitor R:C complex dissociation, cell solution was replaced with 100 μL of CTZ400a-HBSS containing 50 μM Fsk (Calbiochem) and read for an additional 15 cycles. To measure R subunit dimerization, wells were read for 10 cycles after the addition of CTZ400a-HBSS. Luminescence intensities were recorded from each well using the monochromator (400 ± 40 and 400 ± 35 nm) on a Spark 20M fluorescence microplate reader using SparkControl Magellan 1.2 software (TECAN), with a gain of 25 and a 1 s integration time. The interval between cycles was approximately 2 min.

#### Immunoprecipitation and immunoblotting

Immunoprecipitation (IP) was performed from HEK293 cells grown on collagen-coated 6-well plates essentially as described ^112^ using a combination of V5-(LgBiT)-tagged RIβ co-expressed with FLAG-tagged RIβ or EGFP-tagged smAKAP, where indicated. Briefly, cells were transfected with Lipofectamine 2000 and cultured for an additional 36 h. Transfected cells were lysed in IP buffer (20 mM Tris, pH 7.5, 150 mM NaCl, 1% Triton X-100, 1 mM EDTA, 1 mM EGTA, 1 μg/mL leupeptin, 1 mM benzamidine, 2.5 mM Na_4_ P_2_ O_7_, 1 mM Na_3_ VO_4_, and 1 mM β-glycerophosphate) and vortexed briefly to solubilize. Lysates were cleared by centrifugation at 13,000 rpm for 10 min at 4°C. Ten percent of the supernatant was saved as the input, while the remainder was incubated with anti-V5 tag agarose beads (Bethyl Laboratories) for 1 h with rotation at 4° C. Beads were washed four times by centrifugation in IP lysis buffer and eluted by boiling in 2x Laemmli sample buffer for 5 min. Samples were separated on 10% SDS-PAGE gels, transferred to nitrocellulose membrane (GE Healthcare) and immunoblotted. Proteins were visualized using species-specific fluorescent secondary antibodies on an LI-COR Odyssey Classic infrared scanner for dual-color detection using the Odyssey Infrared Imaging System Application Software (Version 3.0.30, LI-COR). Immunoblotting signals were quantified by densitometry using the gel analysis plugin of ImageJ. Co-immunoprecipitation blots are representative of at least three independent experiments.

#### NanoBiT luciferase complementation assays

COS-1 and HEK293 cells were plated at 2.3 × 10^5^ cells per well in collagen-coated (1%) white-walled 96-well plates (Corning), incubated for 16 h, then transfected at the following DNA ratios using Lipofectamine 2000: 20% LgBiT-RIβ, 20% SmBiT-RIβ/RIIα, and 60% pcDNA3.1. After 16–24 h, cells were treated with Opti-MEM for 3 h to deplete basal cAMP levels. Following Opti-MEM incubation, cells were assayed using the Nano-Glo Live Cell Assay System (Promega) on a Cytation 5 imaging reader (BioTek) at 37°C with luminescence detection method and endpoint/kinetic read type. Plates were shaken with linear shake mode for 3 s at a linear frequency of 493 cpm (4 mm), then read using an integration time of 0.1 s at a gain of 200, which was adjusted downward if a saturating signal was reached. Read height of the plate was adjusted based on calibration of luminescent signal using a well from the RIβ^WT^ - LgBiT, RIβ^WT^-SmBiT condition. Each plate was read four times using the reagents provided by the Nano-Glo Live Cell Assay System, followed by another set of four reads following addition of cell-permeable HiBiT peptide, which provides a measure of maximum complementation. Data were double-normalized to maximum complementation and the RIβ^WT^ -LgBiT, RIβ^WT^ -SmBiT condition. Plots of relative normalized luminescence show data from 3 independent experiments with 6–8 technical replicates per experiment.

#### RNA-Seq and RT-PCR

For RNA-Seq and Real-Time (RT)-PCR, RIα KO HEK293T cells were seeded in 6-well plates, then transfected for 24 h with RIβ-m-Ruby2, L50R-mRuby, or mRuby2 control, in triplicate. Cells were washed with ice-cold PBS, then harvested with TRIzol (Invitrogen). Chloroform was added to the cell suspension, followed by centrifugation at 12000 rpm at 4° C for 15 min to separate the RNA-containing supernatant. RNA was then purified using isopropanol, washed with 70% ethanol, and dried. Extracted RNA was dissolved in nuclease-free water and quantified by Nanodrop 2000 (Thermo Scientific). For RNA-Seq, library preparation was performed by Novogene Corporation with 150-bp paired-end reads. For RT-PCR, cDNA was synthesized using PrimeScript RT Master Mix (TaKaRa) using the manufacturer’s recommended conditions. A total of 40 ng of cDNA per reaction was amplified using the iTaq Universal SYBR Green Supermix (Bio-Rad) on a CFX96 Touch Real-Time PCR Detection System (Bio-Rad).

#### Flow cytometry

RIα KO HEK293T cells were transfected at 20–30% confluence for 48 h, with media exchange after 24 h, and harvested by trypsinization with normalization for live cell count using a Countess II cell counter (Life Technologies). Cells were washed and incubated with Annexin V-AF350 conjugate (Invitrogen) in 1x Annexin V binding buffer (10 mM HEPES, pH 7.4, 140 mM NaCl, 25 mM CaCl _2_ ) as directed. Samples of 0.5–1 × 10^5^ cells were run on a Fortessa X20 HTS cytometer using FACSDiva software (BD Biosciences) with similar or identical voltages across replicates. Single-channel and blank samples were included for compensation. Data were processed using FlowJo 10 software (BD Biosciences) using manual gating to exclude cell debris and doublets. Annexin V-positive populations were identified by gating cells with intensities ≥525 and mRuby2 intensities ≥ 10^4^ units. Data points correspond to 3 independent experiments.

#### CRE transcription assays

RIα KO HEK293T cells were seeded at 1.5 × 10^5^ cells per well in 12-well plates and cultured overnight. The following day, cells were transfected with RIβ (WT/R335W), CRE-Luc reporter, and Renilla luciferase plasmids using Lipofectamine 2000. After 24 h, cells were treated with DMSO, 5 μM isoproterenol, or 50 μM forskolin/100 μM 3-isobutyl-1-methylxanthine for 4 h, then assayed using the Dual-Glo Luciferase Assay kit (Promega). Cells were washed with PBS, lysed, and incubated for 15 min at room temperature. Lysates were transferred to a black-walled 96-well plate (Corning). Firefly luciferase activity was measured using a microplate reader (TECAN) at 535–590 nm (central 563 nm), bandwidth 55 nm. STOP & GLO solution was added, and lysates incubated for 10 min in the dark. Renilla luciferase activity was measured at 460–515 nm (central 488 nm), bandwidth 55 nm, with 500 ms integration time. Firefly luciferase activity was normalized to Renilla luciferase activity to control for transfection efficiency. Data points represent individual biological replicates of experimental triplicate.

### QUANTIFICATION AND STATISTICAL ANALYSIS

#### Quantification of cellular puncta

For HEK293T and PC12 cells, puncta per cell were individually quantified by eye on a per-cell basis from epifluorescence or confocal images, excluding puncta which appeared out of plane or irregular in circularity. For primary neurons, maximum intensity projections were analyzed using a custom-written ImageJ macro (Morphometry, https://github.com/ststrack/Strack-Lab-software). Prior to macro analyses, images were denoised using the ImageJ “Unsharp Mask” filter (radius: 2 pixels, weight: 0.6). Morphometry macro parameters were threshold = MaxEntropy, particle size = 4–1600 (pixels), circularity = 0.6–1.0, rolling ball = 20. Output columns selected for display and statistics were “n” (number) and “a” (average puncta size/neuron, converted from pixels to μm^2^ ). Neuronal analyses were blinded to genotype. Figure 5C shows data pooled from two independent neuronal cultures, all others show individual cell data from at least three independent experiments.

#### Calculation of partition coefficients

Integrated intensities of whole-cell ROIs in both RIβ and Cα channels were measured from epifluorescence images, before and after Fsk/IBMX stimulation, using FIJI (ImageJ). Cell ROIs were individually thresholded and partitioning into puncta measured using the Analyze Particles plugin. Partition coefficients were calculated as $\Sigma l_{puncta}/\Sigma l_{cell}$ with respect to the ratio of whole-cell summed intensities of respective channels. Individual cell data were pooled from a minimum of 3 independent experiments and plotted using Prism 10 (GraphPad).

#### Fluorescence recovery after photo bleaching

Data was analyzed as previously described. ^51^ Bleached ROI intensity values were background subtracted and normalized to unbleached reference ROIs to account for any intrinsic photobleaching during acquisition. Corrected values were then normalized to the minimum and maximum intensity values to yield normalized recovery curves between 0 (lowest intensity measurement, the post-bleach timepoint) and 1 (maximum intensity measurement, either the pre-bleach measurement or following a 100% recovery). Resulting values were fit to exponential curves using the ImageJ curve fitting tool. Time to half-maximum recovery $\left({\text{t}}_{1/2}\right)$ values for each sample were calculated from the ImageJ curve-fitting tool using $t_{\frac{1}{2}}=\frac{ln_{f_{o}}(0.5)}{-\tau }$, where τ=b in the exponential recovery equation $y=a{\left(1-e\right)}^{\wedge }(-bx)+c$. Mobile fractions were calculated as the total recovery (a+c). Apparent diffusion coefficients were calculated as $D_{app}=0.224r^{2}/t_{1/2}$ ^113^. Bleached puncta or cytosolic ROIs were pooled from a minimum of 3 independent biological experiments comprising approximately 5–15 bleached samples each. Data were plotted as individual bleached regions using Prism 10 (GraphPad).

#### cAMP response assays

Changes in cellular cAMP were measured using the intensiometric cAMP sensor G-Flamp1, with stimulation using Iso and Fsk/IBMX as indicated. ROIs were drawn in cytosolic or whole-cell regions and background subtracted to calculate fluorescence intensities. Some time courses were plotted as the unnormalized mean response, and others were normalized to the intensity at time zero $\left(I_{0}\right)$, defined as the intensity at the timepoint preceding drug addition, and plotted as time-courses using GraphPad Prism 10 (GraphPad). Time-lapse curves were generated as mean ± 95% CI using pooled data of individual cell traces across ≥ 3 independent biological repeats; dot plots showing puncta and diffuse regions indicate response from the same individual cells.

#### AlphaFold model of full-length RIβ

The full-length human RIβ sequence was subjected to AlphaFold2 in Google Colaboratory (ColabFold 1.5.5) ^107^ using default settings. The best-scoring prediction was selected and compared against published RI crystal structures. Figures were generated using ChimeraX.

#### Disorder and charge predictions of RIβ

Disorder of full-length human RIβ was predicted with IUPred3 (https://iupred3.elte.hu/) ^63^ using the long disorder default. Charge and multivalency predictions for full-length and regions of RIβ were performed via CIDER webserver (http://157.245.85.131:8000/CIDER/analysis/), ^64^ using RIα as a reference.

#### Co-localization and calculation of Pearson’s correlation coefficients

For co-localization analyses, epifluorescence images were analyzed using a custom-written ImageJ macro (colocalizeStack, https://github.com/ststrack/Strack-Lab-software) using default parameters. Images were iteratively deconvolved using a computed point spread function (https://www.optinav.info/Iterative-Deconvolve-3D.html, https://www.optinav.info/Diffraction-Limit-PSF.html). This step was skipped with RIβ WT/mutant puncta colocalization and RIβ colocalization with the broadly nuclear AKAP_nuc_. Pearson’s coefficients were measured from epifluorescence or confocal images, with 5–7 representative images or > 30 cells per experiment, with a minimum of 3 independent experiments each, using the FIJI (ImageJ) Just Another Colocalization Plugin (JACoP) and, for confocal images, with Costes’ automatic threshold with background subtraction.

#### PKA activity assays

Since PKA activity and phase separation are dependent on equilibria between the bound and unbound R:C complex, differences in RIβ-mRuby2 expression levels could affect assay outcome. Cells expressing RIβ-mRuby2 with average whole-cell intensity values between 1,000 and 8,000 units were selected for analysis in all except smAKAP-tethered ExRai-AKAR2 experiments, for which a 3,000-maximum average RIβ intensity was set. ROIs were drawn in cytosolic, nuclear, or whole-cell regions of cells and background subtracted before extracting fluorescence intensities. Excitation ratios (488/380) were calculated for each time point $\left(R\right)$. Some time courses were then normalized to the ratio at time zero $\left(R_{0}\right)$, defined as the ratio at the timepoint preceding drug addition. Where indicated, basal ratios are the unnormalized ratio at time zero. Ratio changes normalized to time zero $\left(\text{Δ}R/R_{0}\right)$ were plotted as time-courses using GraphPad Prism 10 (GraphPad). For Fsk/IBMX stimulation experiments, response amplitude was calculated as the difference between the maximum and basal normalized response for each cell trace $\left(R_{max}-R_{0}\right)$. For Iso stimulation experiments, area under the curve was calculated from normalized time courses using the Prism calculation tool, using a baseline of Y = 0 and ignoring peaks less than 10% of the distance from minimum to maximum Y or any peak defined by fewer than 4 adjacent points. Time-lapse curves were generated as mean ± 95% CI using pooled data of individual cell traces across ≥ 3 independent biological repeats; dot plots indicate response from the same individual cells.

#### Bioluminescence resonance energy transfer assays

BRET emission ratios were calculated as $R=\frac{GFP2}{RLuc8}-c.f.$ for each condition, with the control factor $(c.f.)=\frac{GFP2}{RLuc8}$ in cells expressing only Rluc8. F-induced R:C ratio changes were calculated as $\frac{\text{Δ}R_{F}}{R_{0}}$. Data were plotted in Prism 10 (GraphPad) with time-lapse curves representing mean response ±standard deviation and dots representing individual biological experiments. Plots include data from three biologically independent experiments with three technical replicates per experiment.

#### RNA-Seq analysis

Raw files were uploaded to the Galaxy web platform public server ^106^ for transcript quantification and alignment to the genome using htseq-count and HISAT2. Differential gene expression analysis was conducted in RStudio using DESeq2, ^114^ including quality control, hypothesis testing, and model fitting. Adjusted *p* values less than 0.05 were considered significant. Genes were termed significantly differentially expressed with log_2_ fold change values ≥ |0.5|. Gene set enrichment analysis and single-sample gene set enrichment analysis for the binary RIβ^WT^ vs. L50R comparisons were performed as previously described ^115,116^ using the DESeq2-estimated log fold change values compared to the MSigDB C2 collection of gene sets, ^117^ which contains functional and signaling-related gene sets. Resulting enriched signatures were categorized by known pathways or phenotypes for subsequent validation. The degree of enrichment on a per-sample basis, calculated by ssGSEA, was normalized to the minimum and maximum values and plotted as a heatmap of individual biological replicates per condition. For GSEA, significance was assessed using 10,000 permutations, for ssGSEA 1,000 permutations, and nominal *p* values less than 0.05 were deemed significant. GSEA was conducted in the desktop GSEA software (Broad Institute) and ssGSEA in JupyterLab. Figures were generated with ggplot2 in RStudio and Prism (GraphPad).

#### Real-Time PCR

Gene expression levels were normalized to the expression of *GAPDH* then calculated using the 2^−ΔΔCt^ method. Data points represent individual reactions, with bars showing mean ± standard deviation, and a minimum of four reactions per experimental condition and three per control condition.

#### Neurite outgrowth assays

Dendritic arbors were traced semiautomatically using the ImageJ plugin SNT from maximum intensity projections of stained neurons, and traces were analyzed for length (total pixel number of line traces). For dot plots, approximately 35 cells/experiment were pooled from 3 independent experiments per condition. Analyses were performed blinded to the transfection conditions.

#### Statistics and reproducibility

All data, unless otherwise indicated, were collected from at least three biologically independent experiments. Experiments with data collected on an individual cell basis (*n* = cells), such as puncta quantification and PKA activity assays, were pooled across biologically independent experiments, and where appropriate were plotted to show both population-wide mean response trends as a single curve and single-cell data as dot plots. Other quantitative measurements without single-cell resolution such as BRET, NanoBiT luciferase, and CRE transcription assays were plotted as experimental replicates (*n* = experimental replicates). Statistical analyses were performed in Excel and GraphPad Prism 10 (GraphPad). All data were assessed for normality. Data demonstrating logarithmic distribution were log-transformed before statistical testing. Data with normal distribution prior to or following log-transform were shown as mean ± error, and data without normal distribution were shown as median ± error as specified in figure legends. Comparisons of normally distributed data were conducted as paired or unpaired two-tailed Student’s t-tests for data with equal variance, or with Welch’s correction for unequal variances. Nonparametric data were subject to Wilcoxon tests for paired data or Mann-Whitney tests for unpaired data. Comparisons between three or more groups with normal distribution were conducted using ordinary one-way analysis of variance (ANOVA) followed by post-hoc Tukey’s multiple comparisons test for ordinary data, Dunnett’s multiple comparisons test for ordinary data compared to a defined control or Dunnett’s T3 multiple comparisons test for data with unequal variances. Comparisons between three or more groups of data which were not normally distributed were performed using the Kruskal-Wallis test followed by Dunn’s multiple comparisons test. Statistical significance was set at *p* < 0.05.

## Supplementary Material

1

2

3

4

5

Supplemental information can be found online at https://doi.org/10.1016/j.celrep.2025.115797.

## Figures and Tables

**Table:** KEY RESOURCES TABLE

REAGENT or RESOURCE	SOURCE	IDENTIFIER

Antibodies		

Sheep anti-RIβ polyclonal antibody	R&D Biosystems	Cat#AF4177; RRID: AB_2284184
Donkey anti-sheep polyclonal antibody-AF568	Invitrogen	Cat#A-21099; RRID_10055702
Mouse anti-V5 monoclonal antibody	Invitrogen	Cat#46–0705; RRID: AB_2556564
Goat anti-mouse Atto488	Sigma	Cat #62197; RRID: AB_1137649
Rabbit polyclonal anti-GFP antibody	Abcam	Cat#ab290; RRID: AB_303395
Donkey anti-rabbit IgG (H + L) Alexa Fluor 488	Invitrogen	Cat#A21206; RRID: AB_2535792
Donkey anti-mouse IgG (H + L) Alexa Fluor 555	Invitrogen	Cat#A31570; RRID: AB_2536180
Mouse anti-RI monoclonal antibody	BD Biosciences	Cat#BDB610165; RRID: AB_397567
Mouse anti-PKAc monoclonal antibody	BD Biosciences	Cat#BDB610981; RRID: AB_621842
IRDye 800CW Goat anti-mouse antibody	LI-COR	Cat#926–32210; RRID: AB_621842
IRDye 800CW Goat anti-rabbit antibody	LI-COR	Cat#926–32211; RRID: AB_621843

Bacterial Strains		

DH5α competent bacteria	NEB	Cat#C2987I
TP2000 bacteria	Kim et al.^99^	N/A

Chemicals and Recombinant Proteins		

Forskolin	CalBioChem	Cat#344281
IBMX	Sigma Aldrich	Cat#I7018
Isoproterenol	Sigma Aldrich	Cat#1351005
H89	Cayman Chemical	Cat#10010556
CTZ400a (DeepBlueC)	Nanolight Technology	Cat#340
Q5 High Fidelity Polymerase	NEB	Cat#M0491S
hRIβ pure protein	This paper	N/A
cOmplete^™^ EDTA-free protease inhibitor cocktail	Sigma Aldrich	Cat#COEDTAF-RO
DMEM:F12	ThermoFisher	Cat#12634010
Fetal bovine serum (FBS)	GIBCO	Cat#26140079
Penicillin-streptomycin (Pen-Strep)	GIBCO	Cat#15140122
PolyJet	Signagen	Cat#SL100688
Lipofectamine 2000	Invitrogen	Cat#11668–019
Poly-D-Lysine	Sigma Aldrich	Cat#P6407
Bovine serum albumin (BSA)	Roche	Cat#10738328103
Poly-L-Lysine	Sigma Aldrich	Cat#P1524–1G
Laminin	GIBCO	Cat#23017–015
Trypsin	AMRESCO	Cat#0458–25G
Ethanol 190 Proof	Decon Labs, Inc.	Cat#2801
Collagen Type I, Rat Tail	Corning	Cat#354236
Trypsin	AMRESCO	Cat#0458–25G
ProLong antifade mountant with NucBlue	Invitrogen	Cat#P36981
Annexin V-AF350	Invitrogen	Cat#A23202

Critical Commercial Assays		

HiFi DNA Assembly Kit	NEB	Cat#E5520S
SuperScript III One-step RT-PCR Kit	Invitrogen	Cat#12574–018
Nano-Glo Live Cell Assay System	Promega	Cat#N2012
Dual-Glo Luciferase Assay Kit	Promega	Cat#E2920
RedDot2 far-red nuclear stain	Biotium	Cat#40061

Deposited Data		

RIβ overexpressed RNA-Seq	This paper	GEO: GSE278676
RIβ holoenzyme crystal structure	Ilouz et al.^21^	PDB: 4DIN
cAMP-bound RI(alpha) crystal structure	Bruystens et al.^32^	PDB: 4MX3

Experimental Models: Cell Lines		

HEK293T (Female)	ATCC	Cat#CRL-11268
293T-RIα KO (Female)	Zhang et al.^51^	N/A
HeLa (Female)	ATCC	Cat#CCL-2
PC12 (Male)	Greene & Tischler ^100^	N/A
COS1 (Male)	ATCC	Cat#CRL-1650

Experimental Models: Organisms		

C57BL/6J mice (Male and Female)	The Jackson Laboratory	Strain #: 000664, RRID: IMSR_JAX:000664 https://www.jax.org/strain/000664#

Oligonucleotides		

Primers for construction of recombinant DNA, see Table S3	This paper	N/A
Primers for RT-PCR, see Table S3	This paper	N/A

Recombinant DNA		

pcDNA3.1 RIα-GFP2	Day et al.^101^	N/A
pcDNA3.1 RIβ-GFP2	This paper	N/A
pCAG-G-Flamp1	Wang et al. ^53^	Addgene plasmid #188567
pcDNA3.1 RIβ-G-Flamp1	This paper	N/A
pcDNA3.1 RIβ^E202A^-GFP2	This paper	N/A
pcDNA3.1 RIβ^E326A^-GFP2	This paper	N/A
pcDNA3.1 RIβ^E202A/E326A^-GFP2	This paper	N/A
pcDNA3.1 RIβ^R335K^-GFP2	This paper	N/A
pcDNA3.1 RIβ^R335W^-GFP2	This paper	N/A
pcDNA3.1 RIβ^R335W^-mTagBFP2	This paper	N/A
pcDNA3.1 mTagBFP2-RIα	Zhang et al.^51^	N/A
pcDNA3.1 RIβ-mRuby2	This paper	N/A
pcDNA3.1 RIβ^R335W^-mRuby2	This paper	N/A
pcDNA3.1 RIβ^L50R^-mRuby2	This paper	N/A
pcDNA3.1 mCherry-PKA Cα	Day et al.^101^	N/A
pcDNA3.1 GFP2-PKA Cα	Hardy et al.^55^	N/A
pcDNA3.1 RIβ^ΔD/D^-GFP2	This paper	N/A
pcDNA3.1 RIβ^ΔLinker^-GFP2	This paper	N/A
pcDNA3.1 RIβ^ΔN3A^-GFP2	This paper	N/A
pcDNA3.1 RIβ^ΔCNBs^-GFP2	This paper	N/A
pcDNA3.1 RIβ^D/D^-GFP2	This paper	N/A
pcDNA3.1 RIβ^Linker^-GFP2	This paper	N/A
pcDNA3.1 RIβ^ΔD/D+L^-GFP2	This paper	N/A
pEGFP-N1 LCK	Song et al. ^102^	N/A
pEGFP-N1 RIβ	This paper	N/A
pEGFP-N1 RIβ^L50R^	This paper	N/A
pcLgBiT RIβ-V5-His6	This paper	N/A
pcLgBiT RIβ^L50R^-V5-His6	This paper	N/A
pcSmBiT RIβ-FLAG	This paper	N/A
pcSmBiT RIβ^L50R^-FLAG	This paper	N/A
pcSmBiT RIIα-FLAG	This paper	N/A
pC1 RIβ-FLAG	This paper	N/A
pC1 RIβ^L50R^-FLAG	This paper	N/A
pcDNA3.1 RIβ-V5-His6* sh5	This paper	N/A
pcDNA3.1 RIβ^L50R^ -V5-His6* sh5	This paper	N/A
pN1 RIβ-FLuc	This paper	N/A
pcDNA3.1 H1-shRIβ-5	This paper	N/A
pRluc8-N3-hRIα	Isensee et al.^103^	N/A
pRluc8-N3-RIβ	Diskar et al.^104^	N/A
pRluc8-N3-RIβ^L50R^	This paper	N/A
pRluc8-N3-RIβ^R335W^	This paper	N/A
pcDNA3.1 RIβ-V5	This paper	N/A
pEGFP-N1 smAKAP-FLAG-EGFP	Hardy et al.^55^	N/A
pEGFP-N1 smAKAP^L2P2^-FLAG-EGFP	Hardy et al.^55^	N/A
pCMV AKAP_nuc_ -tagBFP	This paper	N/A
pCMV AKAP_nuc_^L65,70P^ -tagBFP	This paper	N/A
pEGFP-N1 rAKAP1(1–524)	Merrill et al.^82^	N/A
pEGFP-N1 rAKAP1(1–524)^L310P,L316P^	Merrill et al.^82^	N/A
pcDNA3.1 ExRai-AKAR2	Zhang et al.^79^	Addgene plasmid #161753
pcDNA3.1 smAKAP-ExRai-AKAR2	This paper	N/A
pcDNA3.1 smAKAP^L2P2^-ExRai-AKAR2	This paper	N/A
pGL3-CRE-Luc (Firefly)	Castellone et al.^105^	Gift of Gutkind lab
Renilla-Luc	Castellone et al.^105^	Gift of Gutkind lab
pcDNA3.1 mRuby2	This paper	N/A

Software and algorithms		

PRISM (10.1.0)	GraphPad	https://www.graphpad.com/scientific-software/prism/
Inkscape (1.2.2)	Inkscape	https://inkscape.org
FIJI (ImageJ)	NIH	https://imagej.net/software/fiji/downloads
METAFLUOR (7.7)	Molecular devices	https://www.moleculardevices.com/products/cellular-imaging-systems/acquisition-and-analysis-software/metamorph-microscopy
Galaxy	Galaxy Community ^106^	usegalaxy.org
RStudio	Posit	https://posit.co/download/rstudio-desktop/
R (4.3.2)	CRAN	https://cran.rstudio.com/
JupyterLab (4.1.0)	Project Jupyter	jupyter.org/install
GSEA	Broad Institute and University of California	https://www.gsea-msigdb.org/gsea/downloads.jsp
IUPred3	Erdős et al. ^63^	https://iupred3.elte.hu/
CIDER	Holehouse et al.^64^	http://157.245.85.131:8000/CIDER/analysis/
ChimeraX	UCSF	cgl.ucsf.edu/chimerax
FlowJo (10.8.1)	BD Biosciences	https://www.flowjo.com/
NIS-Elements (5.21.02)	Nikon Instruments Inc.	https://www.microscope.healthcare.nikon.com/products/software/nis-elements
LAS X (3.6.0.20104)	Leica	https://www.leica-microsystems.com/products/microscope-software/p/leica-las-x-ls/
BZ-X800 Analyzer (1.1.1.8)	Keyence	https://www.keyence.com/products/microscope/fluorescence-microscope/bz-x700/
BZ-X800 Viewer (1.1.1)	Keyence	https://www.keyence.com/products/microscope/fluorescence-microscope/bz-x700/
BD FACSDiva	BD Biosciences	https://www.bdbiosciences.com/en-us/products/software/instrument-software/bd-facsdiva-software
ColabFold (1.5.5)	Mirdita et al. ^107^	https://colab.research.google.com/github/sokrypton/ColabFold/blob/main/AlphaFold2.ipynb
Odyssey Infrared Imaging System Application Software (3.0.30)	LI-COR	https://www.licor.com/bio/support/answer-portal/imaging-systems/odyssey-classic.html
SparkControl Magellan 1.2	TECAN	https://lifesciences.tecan.com/software-magellan

Other		

PageRuler Plus Prestained Protein Ladder, 10 to 180 kDa	Fisher Scientific	Cat#PI26617
10-well 30 μL 4–15% Mini-PROTEAN^®^ TGX^™^ Precast Protein Gel	Bio-Rad	Cat#4561083
Protino Ni-NTA agarose	Macherey-Nagel	Cat#745400.100
PD-10 desalting column	Cytiva	Cat#17085101
Noble Agar	ThermoFisher	Cat#AAJ1090722
10x Phosphate buffered saline (PBS), pH 7.4	GIBCO	Cat#70011069
1x PBS, pH 7.4	GIBCO	Cat#10010–049
Hank’s balanced salt solution (HBSS)	GIBCO	Cat#14065–056
1x DPBS	GIBCO	Cat#14190–144
Opti-MEM	GIBCO	Cat#31985–070
40 μm Nylon Mesh Sterile Cell Strainer	Fisherbrand	Cat#22363547
NeuroMag Transfection Reagent	OZ Biosciences	Cat#NM51000
Super Magnetic Plate	OZ Biosciences	Cat#MF10000
Anti-V5 Tag Agarose Beads	Bethyl Laboratories	Cat#S190–119
HEPES	GIBCO	Cat#15630–080
100X GlutaMax	GIBCO	Cat#25050–061
B27 Plus Supplement	GIBCO	Cat#A35828–01
Neurobasal Plus Medium	GIBCO	Cat#A3582901
High Glucose DMEM	GIBCO	Cat#11965092

## Highlights

- PKA RIβ undergoes phase separation regulated by PKA-Cα, cAMP, and AKAPs
- The RIβ disordered linker plays a distinct role in maintaining liquid-like assemblies
- RIβ mutants linked to neurological disorders show aberrant phase separation behavior
- Pathological RIβ condensates disrupt PKA signaling via distinct mechanisms
