# Supplementary material for: Aberrant phase separation of two PKA RIβ neurological disorder mutants leads to mechanistically distinct signaling deficits
Source: Cell Rep. Author manuscript; Available in PMC 2025 Aug 21. (PMC12369641; doi:10.1016/j.celrep.2025.115797)
Supplement: 1 [file NIHMS2092663-supplement-1.pdf]

Cell Reports, Volume *44*

Supplemental information

Aberrant phase separation of two PKA RIβ neurological disorder mutants leads to mechanistically distinct signaling deficits

Emily H. Pool, Alexander Glebov-McCloud, Ha Neul Lee, Julia C. Hardy, Valeria Pane, Friedrich W. Herberg, Susan S. Taylor, Sohum Mehta, Stefan Strack, and Jin Zhang

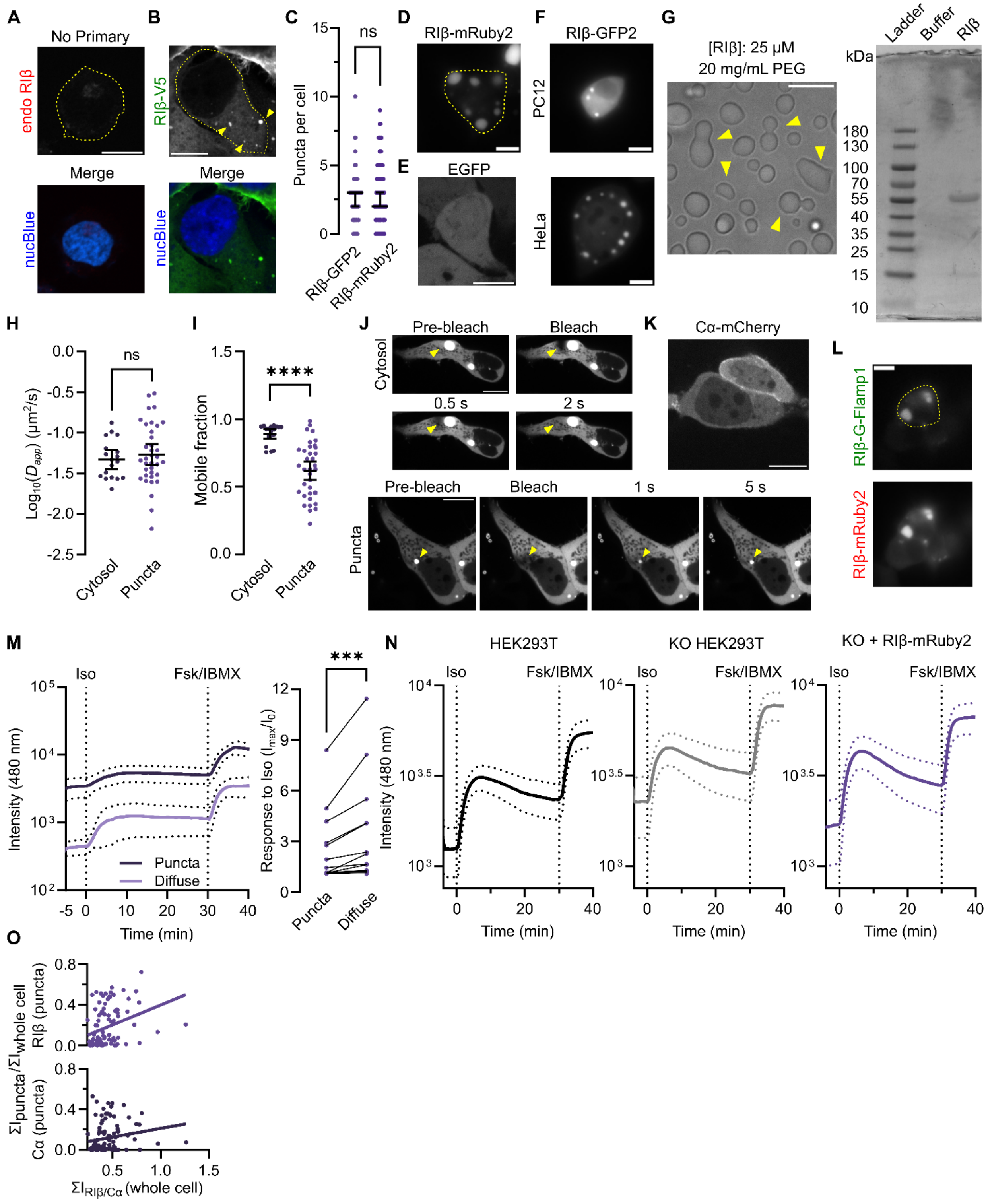

Figure S1. Additional data related to Figure 1. (A) No primary antibody control for RIβ endogenous immunofluorescence in PC12 cells. (B) Representative immunofluorescence image

of HEK293T cells overexpressing RIβ-V5. (C) Quantification of puncta per cell in HEK293T cells singly expressing RIβ-GFP2 or RIβ-mRuby2. $p = 0.303$, Welch's t-test. 95% CI: 2−3 puncta per cell, $n = 97$ cells. (D) Representative image of HEK293T cell overexpressing RIβ-mRuby2, highlighting similar properties to GFP2-tagged RIβ. (E-F) Representative images of RIβ-GFP2 or EGFP controls overexpressed in HEK293T (E), PC12 (F), or HeLa (F) cells, representative of at least 3 independent experiments. (G) (Left) DIC images of *in vitro* droplets of purified 6xHis-RIβ showing surface wetting and droplet fusion. (Right) SDS-PAGE analysis of RIβ purity; expected molecular weight, 46.3 kDa. Buffer, liquid droplet buffer only. (H-J) Metrics ((apparent diffusion coefficients in (H), mobile fractions in (I), and representative time course images (J)) of RIβ-GFP2 FRAP. $p_{Dapp} = 0.457$, 95% CI mobile $\text{fraction}_{\text{puncta}} = 0.462−0.746$, 95% CI $D_{app} = 0.0403–0.0726$ μm^2/s, mobile $\text{fraction}_{\text{cytosol}} = 0.873−0.944$, 95% CI $D_{app} = 0.0357–0.0614$ μm^2/s, ****$p_{\text{mobile fraction}} = 3.79 \times 10^{-9}$, unpaired Welch's t-tests. $n_{\text{puncta}} = 36$, $n_{\text{cytosol}} = 18$ regions. Apparent diffusion coefficients are shown as $\log_{10}$ transform in (H). (K) Cα-mCherry does not form puncta when overexpressed alone in HEK293T cells. (L) Representative epifluorescence images of RIα KO HEK293T cells showing elevated RIβ-G-Flamp1 fluorescence intensity in RIβ-mRuby2-positive condensates. (M) Representative average time-courses of RIα KO HEK293T expressing RIβ-G-Flamp1 showing higher basal fluorescence and lower response to cAMP stimulation from RIβ-G-Flamp1-positive puncta vs. diffuse regions of the same cells. ***$p = 3.66 \times 10^{-4}$, paired Wilcoxon test. $n = 14$ cells from four independent experiments. (N) Untargeted G-Flamp1 shows robust response to cAMP stimulation across multiple cellular models. $n = 57, 54, 63$ cells respectively. (O) Partition coefficients of cAMP-stimulated RIβ-GFP2 or Cα-mCherry puncta versus the ratio of RIβ:Cα summed whole-cell intensities for co-overexpressed HEK293T cells, including cells with partitions = 0 (no puncta),

and linear regression. Scale bars: 10 µm. Dashed lines indicate cell borders. Time-lapse curves in (M) and (N) show mean ± 95% CI. Dot plot error bars in (C) indicate median ± 95% CI, and in (H), (I), mean ± 95% CI.

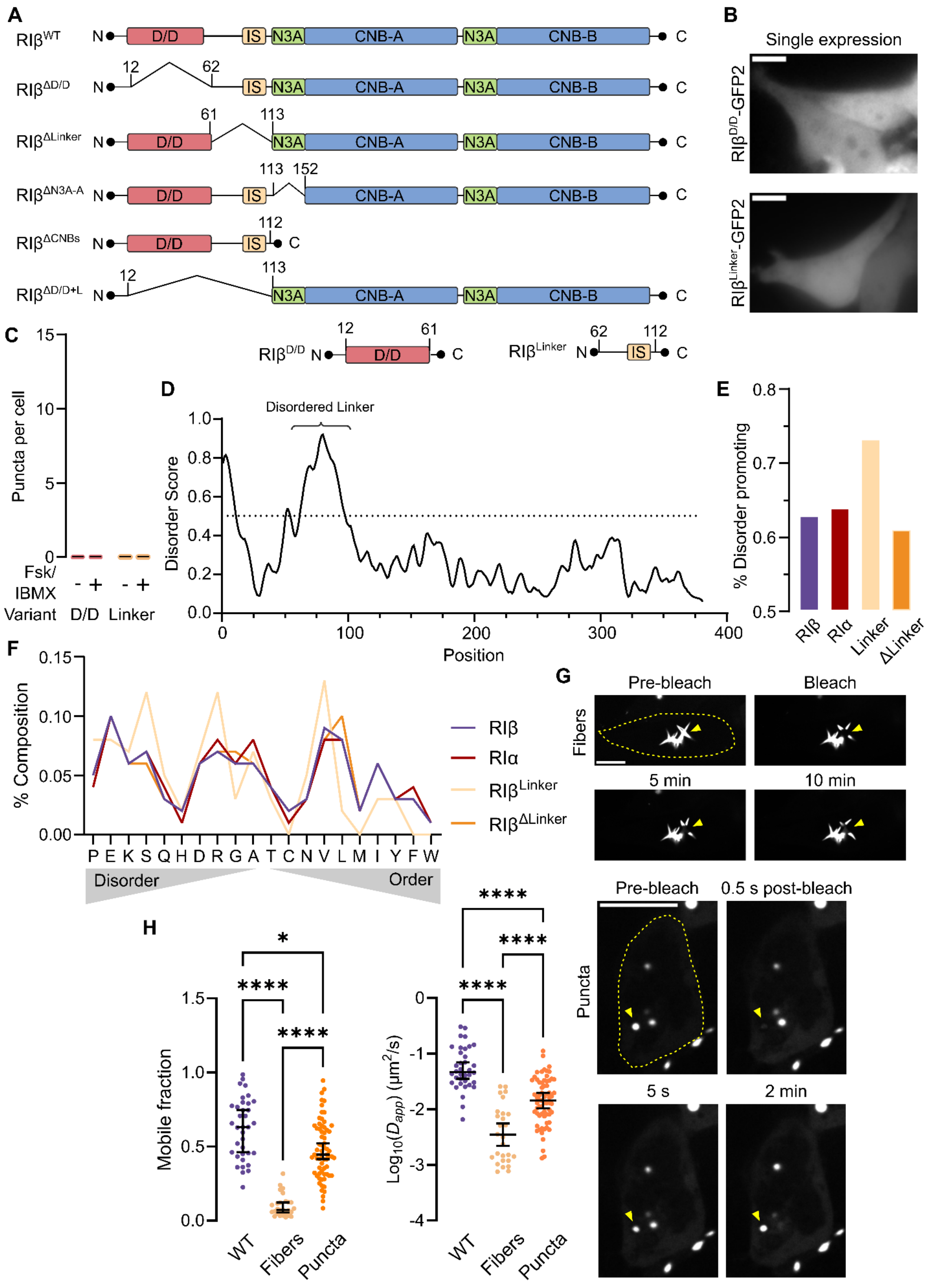

A
RIβWT
N D/D IS N3A CNB-A N3A CNB-B C
RIβΔD/D
12 62
RIβΔLinker
61 113
RIβΔN3A-A
113 152
RIβΔCNBs
112
RIβΔD/D+L
12 113
B
Single expression
RIβD/D-GFP2
RIβLinker-GFP2
RIβD/D N 12 D/D 61 C
RIβLinker N 62 IS 112 C
C
Puncta per cell
Fsk/ IBMX
Variant D/D Linker
D
Disordered Linker
Disorder Score
Position
E
% Disorder promoting
RIβ RIα Linker ΔLinker
F
% Composition
P E K S Q H D R G A T C N V L M I Y F W
Disorder
Order
RIβ
RIα
RIβLinker
RIβΔLinker
G
Pre-bleach
Bleach
Fibers
5 min
10 min
Pre-bleach
0.5 s post-bleach
Puncta
5 s
2 min
H
Mobile fraction
Log10(Dapp) (μm2/s)
WT Fibers Puncta

Figure S2. Additional data related to Figure 2. (A) Schematics of RIβ-GFP2 domain deletion constructs. (B-C) Representative images and puncta quantification of singly expressed regions of RIβ-GFP2. $n_{\mathrm{D/D}} = 78$, $n_{\mathrm{Linker}} = 69$ cells. (D) IUPred3 disorder prediction across full-length RIβ. (E-F) Compositional analysis of the percentage of disorder-promoting residues across different regions of RIβ, compared to full-length RIα, using CIDER. (G) Representative images of RIβ$^{\Delta\mathrm{Linker}}$-GFP2 FRAP of fibrillar structures (top, singly overexpressed) or puncta (bottom, co-expressed with Cα-mCherry). Dashed lines indicate cell borders. (H) Mobile fractions and apparent diffusion coefficients of RIβ$^{\Delta\mathrm{Linker}}$-GFP2 assemblies. Fibrillar assemblies: 95% CI $D_{app} = 0.00220–0.00558\ \mu m^2/s$, mobile fraction = 0.0558–0.123, $n = 27$ ROIs plus three ROIs which did not recover [DNR]. Puncta: 95% CI $D_{app} = 0.0110–0.0178\ \mu m^2/s$, mobile fraction = 0.415−0.522, $n = 66$ puncta. Mobile fractions: ****$p_{\mathrm{WT/fibers}} = 6 \times 10^{-15}$, *$p_{\mathrm{WT/puncta}} = 0.0248$, ****$p_{\mathrm{fibers/puncta}} = 3.02 \times 10^{-10}$. Log-transformed D_{app}: ****$p_{\mathrm{WT/fibers}} < 1 \times 10^{-15}$, ****$p_{\mathrm{WT/puncta}} = 8.44 \times 10^{-10}$, ****$p_{\mathrm{fibers/puncta}} = 9.00 \times 10^{-6}$. Wild-type data reproduced from Figure S1. Dot plot error bars indicate median ± 95% CI in (C) and (H) (left) or mean ± 95% CI in (H) (right). Statistical significance of mobile fractions in (H) indicated by Kruskal-Wallis test and of D_{app} by Brown-Forsythe and Welch's ANOVA with multiple comparisons. Scale bars = 10 μm. Data are from at least three independent experiments.

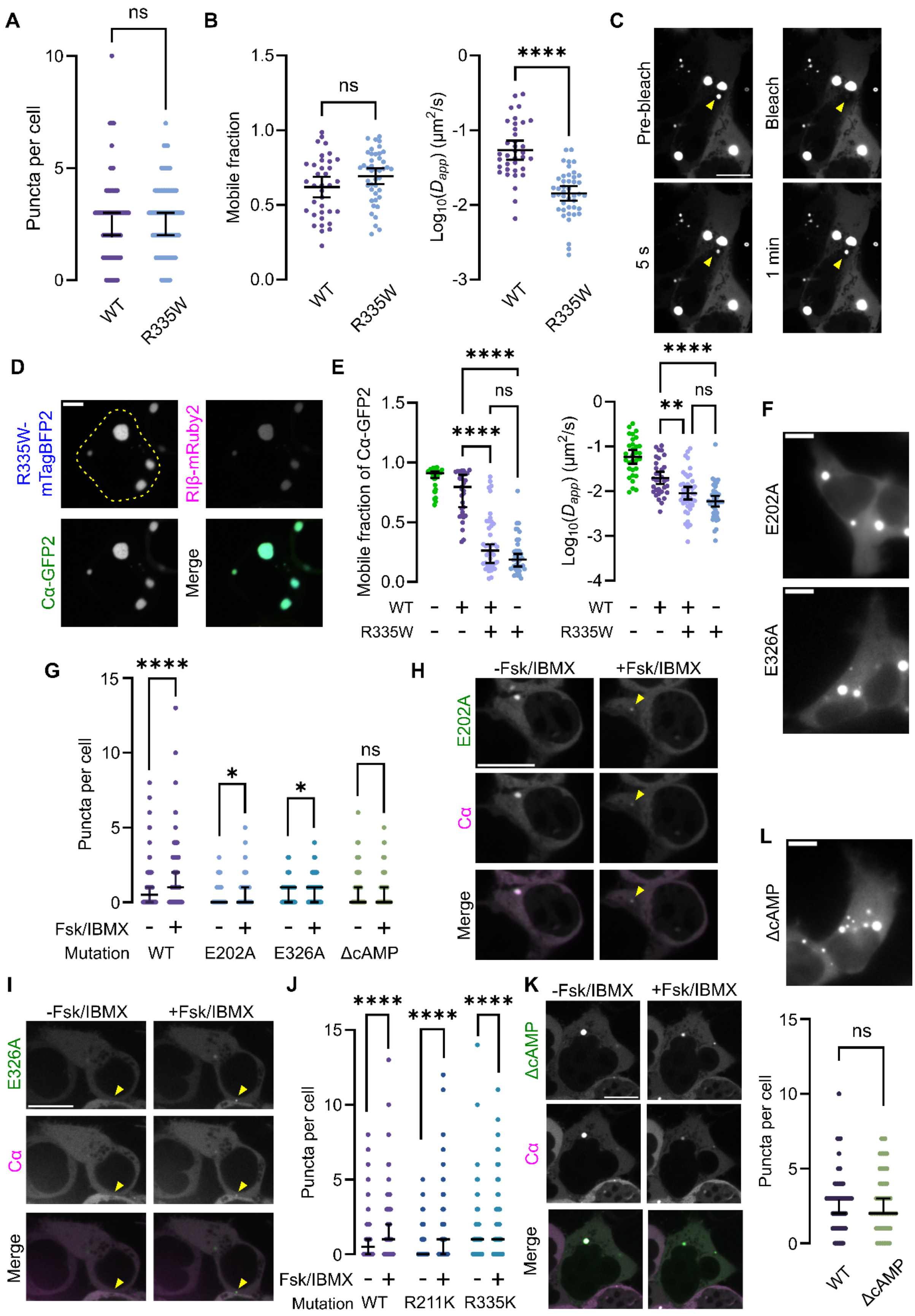

A
B
C
D
E
F
G
H
I
J
K
L
Puncta per cell
WT
R335W
ns
Mobile fraction
Log10(Dapp) (µm2/s)
****
Pre-bleach
Bleach
5 s
1 min
R335W-mTagBFP2
RIβ-mRuby2
Cα-GFP2
Merge
Mobile fraction of Cα-GFP2
**
E202A
E326A
Fsk/IBMX
Mutation
ΔcAMP
*
-Fsk/IBMX
+Fsk/IBMX
Cα
R211K
R335K

Figure S3. Additional data related to Figure 3. (A) Quantification of puncta per cell with RIβ-GFP2 or R335W singly expressed in HEK293T. 95% CI: 2−3, p = 0.707, unpaired Welch's t-test, n_{R335W} = 138 cells. (B) RIβ^{R335W} FRAP mobile fraction and log_{10} transform of D_{app} values compared to RIβ^{WT}, n_{R335W} = 43 puncta, $p_{mobile\ fraction}$ = 0.0903, ****p_{Dapp} = 1.39 × 10^{-10}, unpaired t-tests. (C) Representative time-lapse images of RIβ^{R335W} FRAP. (D) Representative confocal images of co-localized Cα-GFP2 in RIβ-mRuby2/RIβ^{R335W}-mTagBFP2 co-condensates (transfected 2:1:1, respectively). (E) Quantification of Cα recovery from bleached Cα/RIβ/RIβ^{R335W} co-condensates as in (D) or Cα/RIβ^{R335W} condensates, compared to bleached Cα in RIβ^{WT} condensates. Recovery of diffuse Cα-GFP2 is shown as a control (green). n_{WT} = 33, n_{R335W} = 42, $n_{WT/R335W}$ = 43 puncta, $n_{C\alpha}$ = 31 ROIs. Mobile fractions: ****$p_{WT/R335W}$ = 1.76 × 10^{-13}, ****$p_{WT/WT+R335W}$ = 2.67 × 10^{-8}, $p_{R335W/WT+R335W}$ = 0.165 by Kruskal-Wallis test; apparent diffusion coefficients: ****$p_{WT/R335W}$ = 9. 12 × 10^{-7}, **$p_{WT/WT+R335W}$ = 0.00147, $p_{R335W/WT+R335W}$ = 0.113 by ordinary one-way ANOVA. (F) Representative images of RIβ^{E202A} or RIβ^{E326A}-GFP2 overexpressed alone. (G) Quantification of puncta per cell in Fsk/IBMX-stimulated cells co-overexpressing wild-type, RIβ^{E202A}, RIβ^{E326A}, or RIβ$^{\Delta cAMP}$-GFP2 with Cα-mCherry. Basal: 95% CI_{E202A}: 0−0, cAMP-stimulated: 95% CI_{E202A}: 0−1, 95% $CI_{E326A\pm Fsk/IBMX}$: 0−1, 95% $CI_{\Delta cAMP\pm Fsk/IBMX}$ = 0−1 puncta per cell; n_{WT} = 70, n_{E202A} = 87, n_{E326A} = 98, $n_{\Delta cAMP}$ = 112 cells. ****p_{WT} = 2.07 × 10^{-6}, *p_{E202A} = 0.0434, *p_{E326A} = 0.0181, $p_{\Delta cAMP}$ = 0.202, Wilcoxon tests. (H-I) Representative epifluorescence images from experiments in (G). Yellow arrowheads indicate puncta. (J) Quantification of puncta per cell in Fsk/IBMX-stimulated cells co-overexpressing RIβ^{R211K} or RIβ^{R335K}-GFP2 with Cα-mCherry. R211K 95% CI_{basal} = 0−0, 95% CI_{+cAMP} = 0−1; 95% $CI_{R335K\pm cAMP}$ = 1−1; ****p_{R211K} = 1.69 × 10^{-6}, ****p_{R335K} = 1.24 × 10^{-5}, Wilcoxon test, n_{R211K} = 104, n_{R335K} = 175 cells. (K) Representative images of RIβ$^{\Delta cAMP}$-GFP2 co-overexpressed

with Cα-mCherry and stimulated with Fsk/IBMX for 20 min. Quantification shown in Figure S3G. (L) Quantification of puncta per cell with RIβ^{WT} or RIβ$^{\Delta cAMP}$-GFP2 singly expressed. 95% CI: 2−3 puncta per cell, *n* = 59 cells; *p* = 0.705, unpaired Welch's t-test. Scale bars: 10 µm except (D), 5 µm. Dot plots show median ± 95% CI except (B) and (E), mean ± 95% CI. Experiments conducted in HEK293T cells except WT/R335W co-condensates in (D-E), in RIα KO HEK293T. Wild-type data reproduced from Figure 1 and Figure S1.

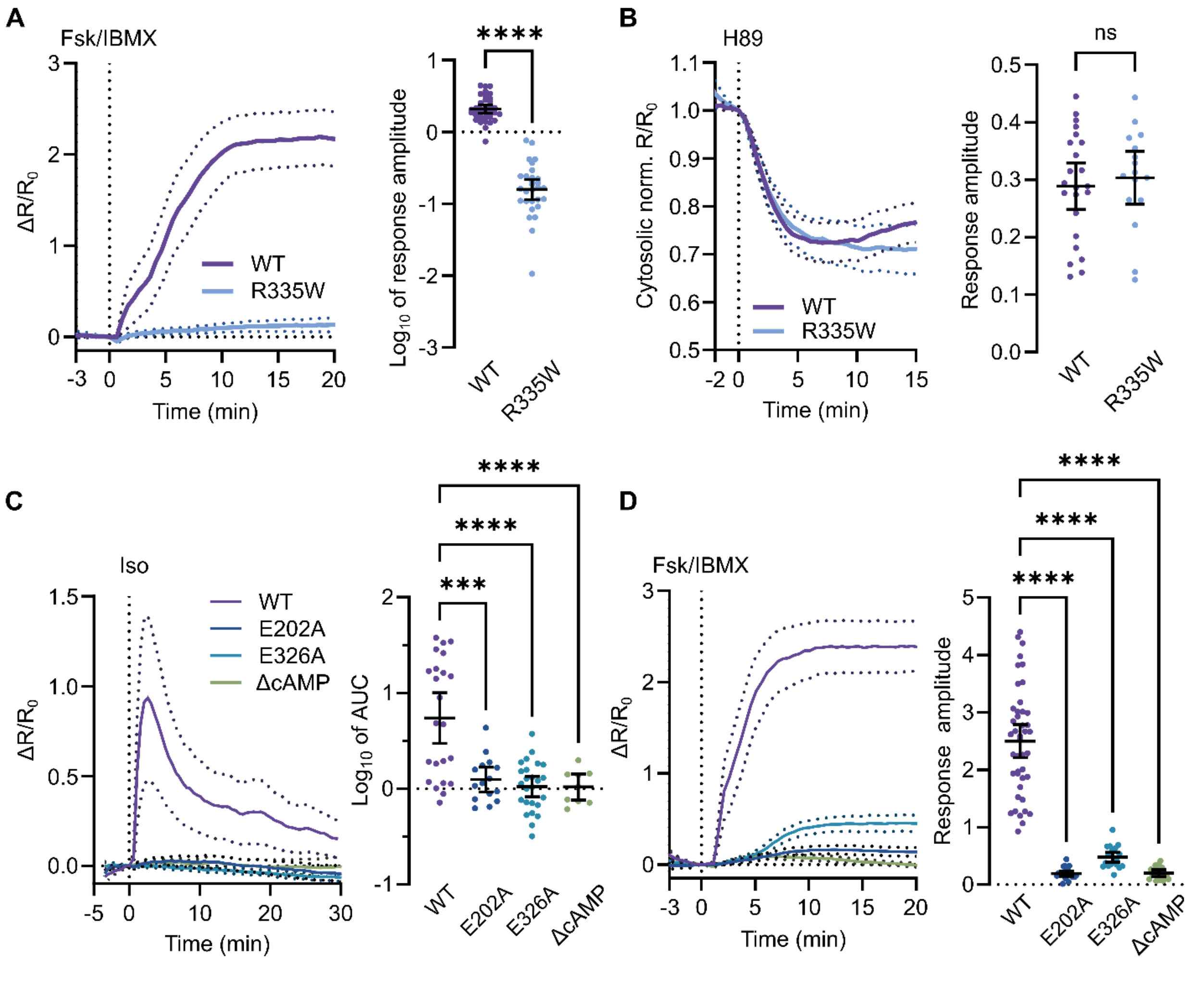

Figure S4. Supplementary data related to Figure 4. (A) Normalized ExRai-AKAR2 response curves in Fsk/IBMX-stimulated cells co-expressing RIβ-mRuby2 or variant and $\log_{10}$ transform of response amplitudes. Untransformed 95% CI of median response amplitude $\Delta R/R_0$ RIβ^{WT} = 1.76–2.36, RIβ^{R335W} = 0.109–0.231, n = 36, 29 cells, respectively. ****$p < 1 \times 10^{-15}$, unpaired Welch's t-test. (B) Normalized basal cytosolic PKA activity for wild-type and R335W-expressing cells measured as change in PKA activity following inhibition with 20 µM H89. n_{WT}= 23, n_{R335W} = 16 cells. p = 0.622, unpaired t-test. (C) Normalized PKA activity response to 5 µM Iso in cells overexpressing wild-type or CNB mutant RIβ-mRuby2 and $\log_{10}$ transformed area under the curve. ***$p_{WT\ vs\ E202A} = 2.32 \times 10^{-4}$, ****$p_{WT\ vs\ E326A} = 4.32 \times 10^{-5}$, ****$p_{WT\ vs\ \Delta cAMP}$ =

5.43×10^{-5}. Untransformed 95% CI of median AUC WT = 1.83−16.9, E202A = 0.778−1.75, E326A = 0.718−1.52, ΔcAMP = 0.716−1.44; *n* = 23, 15, 25, 9 cells, respectively. (D) Normalized PKA activity response to Fsk/IBMX in cells overexpressing CNB mutant RIβ-mRuby2. ****$p_{all} < 1 \times 10^{-15}$. 95% CI of mean response amplitude WT = 2.21−2.79, E202A = 0.147−0.241, E326A = 0.393−0.566, ΔcAMP = 0.142−0.260; *n* = 43, 20, 20, 17 cells, respectively. Plots show mean ± 95% CI. Experiments conducted in RIα KO HEK293T cells. Multiple comparisons performed via Brown-Forsythe and Welch ANOVA with Dunnett's T3 multiple comparisons, with respect to RIβ wild-type.

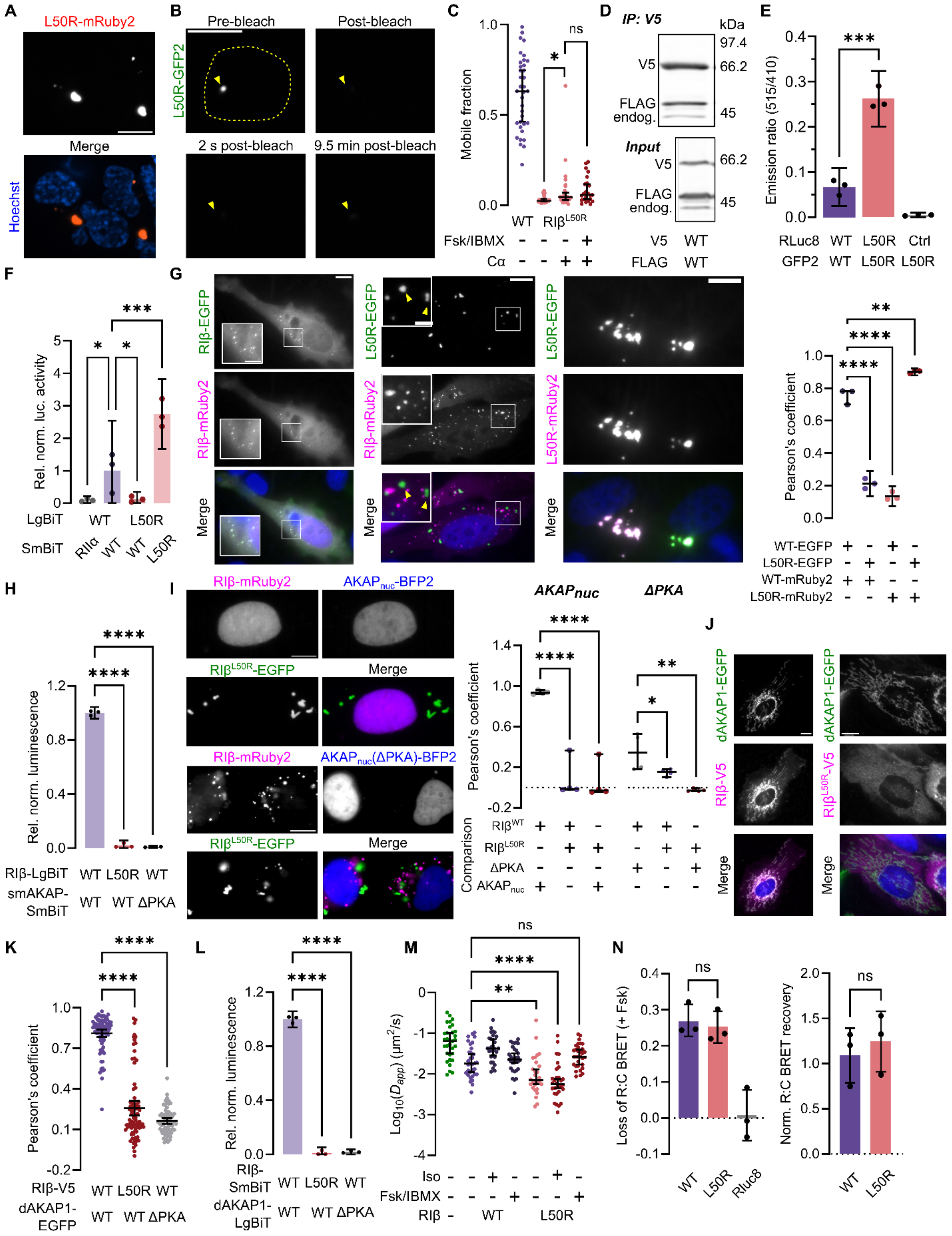
A
L50R-mRuby2
Merge
Hoechst
B
Pre-bleach
Post-bleach
L50R-GFP2
2 s post-bleach
9.5 min post-bleach
C
Mobile fraction
ns
WT
RIβ^L50R
Fsk/IBMX
Cα
D
IP: V5
kDa
97.4
66.2
45
V5
FLAG endog.
Input
E
Emission ratio (515/410)
RLuc8
GFP2
WT
L50R
Ctrl
F
Rel. norm. luc. activity
LgBiT
SmBiT
RIIα
G
RIβ-EGFP
RIβ-mRuby2
Merge
L50R-EGFP
L50R-mRuby2
Pearson's coefficient
WT-EGFP
WT-mRuby2
H
Rel. norm. luminescence
RIβ-LgBiT
smAKAP-SmBiT
ΔPKA
I
AKAPnuc-BFP2
RIβ^L50R-EGFP
AKAPnuc(ΔPKA)-BFP2
AKAPnuc
Comparison
RIβ^WT
J
dAKAP1-EGFP
RIβ-V5
RIβ^L50R-V5
K
RIβ-V5
dAKAP1-EGFP
L
RIβ-SmBiT
dAKAP1-LgBiT
M
Log10(Dapp) (μm²/s)
Iso
RIβ
N
Loss of R:C BRET (+ Fsk)
Rluc8
Norm. R:C BRET recovery

Figure S5. Supplementary information related to Figure 5. (A) Representative image of L50R-mRuby2 expression in HEK293T cells co-stained with Hoechst. (B) Representative photobleached punctum of L50R-GFP2 expressed alone. (C) Mobile fractions of bleached WT- and RIβ^{L50R}-GFP2 condensates with or without Cα-mCherry and cAMP stimulation. *$p_{\pm C\alpha}$ = 0.0186, $p_{\pm cAMP}$ = 0.387, Mann-Whitney tests; $n_{L50R\ basal}$ = 23 + 11 DNR, $n_{+C\alpha}$ = 26, n_{+cAMP} = 23 + 4 DNR condensates. See also Figure 5C. (D-F) Representative control WT/WT co-IP blot (D), (E) BRET, and (F) NanoBiT luciferase complementation to evaluate RIβ^{L50R} self-association; see also Figure 5E. BRET: ****p* = 0.000351, unpaired Student's t-test. NanoBiT luciferase complementation with RIIα:WT as a negative control: ***$p_{WT/WT\ vs.\ L50R/L50R}$ = 0.000455, *$p_{WT/WT\ vs.\ WT/RII\alpha}$ = 0.0354, *$p_{WT/WT\ vs.\ L50R/WT}$ = 0.0410. *n* = 3 independent experiments with 8 technical replicates per experiment. Plots show experimental means and 95% CI. (G) Images of HeLa cells showing RIβ^{WT} and RIβ^{L50R} form distinct, exclusive condensates. Dot plot indicates mean Pearson coefficients ± 95% CI; *n* = 3 experiments with approximately 30 cells/experiment/condition. ****$p_{L50R/WT}$ = 6.82 × 10^{-8}, ****$p_{WT/L50R}$ = 2.34 × 10^{-8}, **$p_{L50R/L50R}$ = 0.00110, one-way ANOVA with Dunnett's multiple comparison test. (H) Luciferase complementation between smAKAP and RIβ validating the lack of RIβ^{L50R}:smAKAP interaction. ****$p_{L50R/smAKAP}$ = 2.49 × 10^{-10}, ****$p_{WT/\Delta PKA}$ = 2.36 × 10^{-10}. Data are from 3 independent experiments with 6 technical replicates per experiment. (I) Images of HeLa cells co-expressing RIβ-mRuby2, RIβ^{L50R}-EGFP, and AKAPnuc$^{WT/\Delta PKA}$-BFP2. Representative of four experiments, *n* = 32-49 cells/experiment/condition. Graphs show average Pearson coefficients of four experiments. AKAP$_{nuc}$ (left): ****$p_{WT/L50R}$ = 4.33 × 10^{-5}, ****$p_{L50R+AKAP}$ = 3.53 × 10^{-5}; right AKAP$_{nuc}^{\Delta PKA}$ (right): *$p_{WT/L50R}$ = 0.0478, **$p_{L50R+\Delta PKA}$ = 0.00160 by one-way ANOVAs with Dunnett's multiple comparisons tests. (J) Images of HeLa cells co-expressing RIβ- or RIβ^{L50R}-V5

and dAKAP1-EGFP. *n* = 2 independent experiments with >30 cells/experiment/condition. (K) Individual cell Pearson's coefficients from (J). ****$p_{\text{all}} < 1 \times 10^{-15}$, Brown-Forsythe and Welch ANOVA with Dunnett's T3 multiple comparisons test. (L) Luciferase complementation between dAKAP1 and RIβ validating the lack of RIβ$^{\text{L50R}}$:dAKAP1 binding. ****$p_{\text{L50R/dAKAP1}} = 1.09 \times 10^{-9}$, ****$p_{\text{WT/}\Delta\text{PKA}} = 1.14 \times 10^{-9}$. Data from 3 independent experiments with 6 technical replicates per experiment. (M) Log_{10} transform of apparent diffusion coefficients of Cα-GFP2 in WT and RIβ$^{\text{L50R}}$ puncta. **$p_{\text{WT/L50R}} = 0.00564$, ****$p_{\text{L50R+Iso}} = 1.56 \times 10^{-5}$, $p_{\text{L50R+Fsk/IBMX}} = 0.338$, ordinary one-way ANOVA with Dunnett's multiple comparisons test, using RIβ^{WT} as the reference. Bleached diffuse Cα-GFP2 control (green) reproduced from Figure S3, *n* = 31, 33, 29, 31, 28, 30, 32 ROIs from left to right and a minimum of 3 independent experiments. (N) Change in R:C BRET emission ratio upon forskolin stimulation and recovery following forskolin wash-out. $p_{\text{dissociation}} = 0.633$, $p_{\text{reassociation}} = 0.588$, unpaired Student's t-test. Scale bars: 10 µm; insets, 3 µm. Yellow arrowheads indicate puncta, dashed yellow lines indicate cell outlines. NanoBiT complementation data in (F), (H), and (L) were assessed by one-way ANOVA with Dunnett's multiple comparisons tests. Data in (C), (I) are shown as median ± 95% CI, in (E-H), (K-M) are mean ± 95% CI, and in (E) and (N) are mean ± SD. Data in (A-D), (H), (L-M) acquired in HEK293 cells, (F) in COS-1, (G), (I-K) in HeLa cells, and (E) and (N) in RIα KO HEK293T. Representative image brightness in (A) (Hoechst only), (G), (I), and (J) adjusted for visual clarity. Wild-type data in (C) and (M) reproduced from Figures 1 and S1.

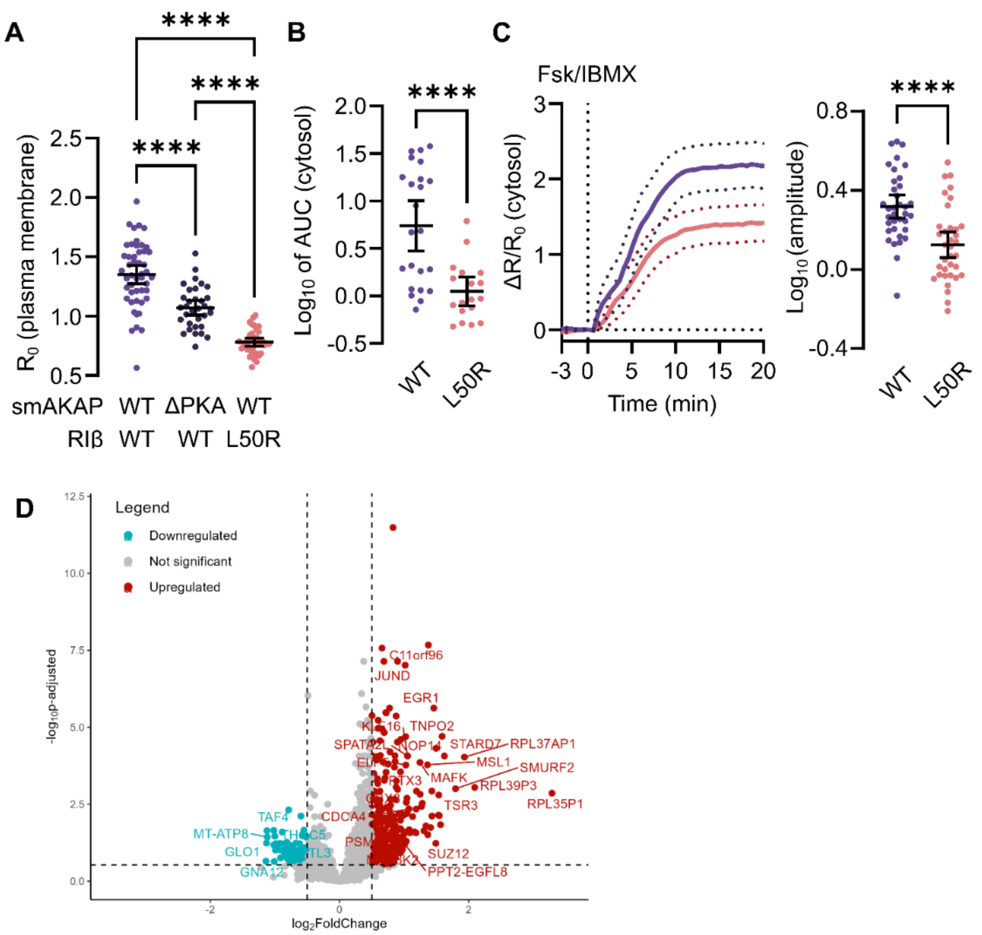

Figure S6. Supplementary data related to Figure 6. (A) Basal plasma membrane-localized PKA activity of RIα KO HEK293T cells overexpressing $RI\beta^{WT}$- or $RI\beta^{L50R}$-mRuby2, measured as the average baseline signal of smAKAP-ExRai-AKAR2. $****p_{\text{WT vs L2P2}} = 4.33 \times 10^{-7}$, $****p_{\text{others}} < 1 \times 10^{-15}$, Brown-Forsythe and Welch ANOVA. $n_{\text{WT}} = 50$, $n_{\text{L2P2}} = 34$, $n_{\text{L50R}} = 36$ cells. (B) Quantification of total PKA activity from curves in Figure 6D. $****p = 4.20 \times 10^{-5}$, Welch's t-test, $n_{\text{WT}} = 23$, $n_{\text{L50R}} = 18$ cells. (C) Cytosolic PKA response in cells overexpressing $RI\beta^{WT}$- or $RI\beta^{L50R}$-mRuby2 stimulated with Fsk/IBMX for 20 min. 95% CI of $\log_{10}$ mean response amplitude WT = 0.259−0.375, L50R = 0.0594−0.190, $****p = 2.83 \times 10^{-5}$, unpaired t-test, $n_{\text{WT}} = 36$, $n_{\text{L50R}} = 35$ cells. (D) Volcano plot of significant DEGs in $RI\beta^{L50R}$ vs wild-type RNA-Seq as identified by DESeq2. Figure generated with ggplot2 in RStudio 4.3.2. For (A-C),

data are mean ± 95% CI. Experiments conducted in RIα KO HEK293T cells. Wild-type data in (B-C) reproduced from Figure 4 and Supplementary S4.
